# Financial Relationships between Organizations That Produce Clinical Practice Guidelines and the Biomedical Industry: A Cross-Sectional Study

**DOI:** 10.1371/journal.pmed.1002029

**Published:** 2016-05-31

**Authors:** Paul Campsall, Kate Colizza, Sharon Straus, Henry T. Stelfox

**Affiliations:** 1 Department of Critical Care Medicine, University of Calgary, Calgary, Alberta, Canada; 2 Department of Medicine, University of Calgary, Calgary, Alberta, Canada; 3 Department of Medicine, Li Ka Shing Knowledge Institute, St. Michael’s Hospital, University of Toronto, Toronto, Ontario, Canada; 4 Department of Critical Care Medicine, University of Calgary, Calgary, Alberta, Canada; 5 Department of Community Health Sciences, University of Calgary, Calgary, Alberta, Canada; 6 Department of Medicine, University of Calgary, Calgary, Alberta, Canada; 7 O’Brien Institute for Public Health, University of Calgary, Calgary, Alberta, Canada; 8 Alberta Health Services, Calgary, Alberta, Canada; National Institutes of Health, UNITED STATES

## Abstract

**Background:**

Financial relationships between organizations that produce clinical practice guidelines and biomedical companies are vulnerable to conflicts of interest. We sought to determine whether organizations that produce clinical practice guidelines have financial relationships with biomedical companies and whether there are associations between organizations’ conflict of interest policies and recommendations and disclosures provided in guidelines.

**Methods and Findings:**

We conducted a cross-sectional survey and review of websites of 95 national/international medical organizations that produced 290 clinical practice guidelines published on the National Guideline Clearinghouse website from January 1 to December 31, 2012. Survey responses were available for 68% (65/95) of organizations (167/290 guidelines, 58%), and websites were reviewed for 100% (95/95) of organizations (290/290 guidelines, 100%). In all, 63% (60/95) of organizations producing clinical practice guidelines reported receiving funds from a biomedical company; 80% (76/95) of organizations reported having a policy for managing conflicts of interest. Disclosure statements (disclosing presence or absence of financial relationships with biomedical companies) were available in 65% (188/290) of clinical practice guidelines for direct funding sources to produce the guideline, 51% (147/290) for financial relationships of the guideline committee members, and 1% (4/290) for financial relationships of the organizations producing the guidelines. Among all guidelines, 6% (18/290) disclosed direct funding by biomedical companies, 40% (117/290) disclosed financial relationships between committee members and biomedical companies (38% of guideline committee members, 773/2,043), and 1% (4/290) disclosed financial relationships between the organizations producing the guidelines and biomedical companies. In the survey responses, 60 organizations reported the procedures that they included in their conflict of interest policies (158 guidelines): guidelines produced by organizations reporting more comprehensive conflict of interest policies (per additional procedure, range 5–17) included fewer positive (rate ratio [RR] 0.91, 95% CI 0.86–0.95) and more negative (RR 1.32, 95% CI 1.09–1.60) recommendations regarding patented biomedical products. The clinical practice guidelines produced by organizations reporting more comprehensive conflict of interest policies were also more likely to include disclosure statements for direct funding sources (odds ratio [OR] 1.31, 95% CI 1.10–1.56) and financial relationships of guideline committee members (OR 1.36, 95% CI 1.09–1.79), but not financial relationships of the organizations (0 disclosures). Limitations of the study include the use of the National Guideline Clearinghouse as the single source of clinical practice guidelines and the self-report of survey responses and organizations’ website postings.

**Conclusions:**

Financial relationships between organizations that produce clinical practice guidelines and biomedical companies are common and infrequently disclosed in guidelines. Our study highlights the need for an effective policy to manage organizational conflicts of interest and disclosure of financial relationships.

## Abstract 2

In this cross sectional study, Henry Stelfox and colleagues examine conflict of interest policies for organizations that produce clinical practice guidelines disclosing their relationships with biomedical companies.

## Author Summary

**Why Was This Study Done?:**

- Clinical practice guidelines are designed to influence the practice of large numbers of providers and are vulnerable to influence from biomedical companies.
- To manage conflicts of interest, the disclosure of financial relationships with biomedical companies is recommended for individuals and organizations involved in producing clinical practice guidelines.
- It is unknown whether organizations that produce clinical practice guidelines have financial relationships with biomedical companies, whether they disclose these relationships, and what policies they use to manage conflicts of interest.

**What Did the Researchers Do and Find?:**

- The majority of organizations (63%) that published clinical practice guidelines on the National Guideline Clearinghouse website in 2012 reported receiving funds from biomedical companies on their website or in response to a survey.
- Very few (1%) of the published clinical practice guidelines disclosed financial relationships between the organizations producing the clinical practice guidelines and biomedical companies.
- Clinical practice guidelines produced by organizations with more comprehensive conflict of interest policies included relatively fewer positive (−9%) and more negative (+32%) recommendations regarding patented biomedical products and were more likely to include disclosure statements for direct funding sources (+31%) and the financial relationships of the guideline committee members (+36%).

**What Do These Findings Mean?:**

- Effective policies for managing conflicts of interest for organizations that produce clinical practice guidelines are needed.
- We support complete disclosure of financial relationships with biomedical companies for organizations that produce clinical practice guidelines.

## Introduction

Financial relationships between biomedical companies and the individuals and organizations involved in the production of clinical practice guidelines are vulnerable to conflicts of interest [1–5]. These types of relationships can have undue influence because clinical practice guidelines are resource intensive to produce [6] and are developed by a small number of expert clinicians who determine the scope of the guidelines, synthesize and interpret the published evidence base, and provide recommendations. The potential impacts of conflicts of interest are large because clinical practice guidelines are designed to be widely disseminated and influence the practice patterns of large numbers of healthcare providers [1,7,8].

To help manage conflicts of interest, the Institute of Medicine Committee on Conflict of Interest in Medical Research, Education, and Practice recommended that all sources of funding, both direct and indirect, be “publicly disclose[d] with each guideline” [3]. Organizations that produce clinical practice guidelines and journals that publish guidelines have responded by requiring disclosure statements from experts who participate in guideline development [9]. However, it is unclear whether organizations that produce clinical practice guidelines have financial relationships with the biomedical industry, whether they have policies to manage conflicts of interest, and whether financial relationships are disclosed within guidelines.

We therefore conducted an observational study to determine whether organizations that produce clinical practice guidelines receive funds from biomedical companies, what policies (and specific procedures) they use to minimize and/or manage conflicts of interest, what disclosures they provide within guidelines, and whether there are associations between organizations’ conflict of interest policies and the recommendations and disclosures provided in guidelines.

## Methods

We employed the conceptualization of conflict of interest developed by Emanuel and Thompson [10] and defined a conflict of interest as “a set of conditions in which professional judgment concerning a primary interest (such as a patient’s welfare or the validity of research) tends to be unduly influenced by a secondary interest (such as financial gain)” [11]. We used the National Guideline Clearinghouse definition of a clinical practice guideline: “clinical practice guidelines are systematically developed statements to assist practitioner and patient decisions about appropriate health care for specific clinical circumstances” [12,13]. The study was conducted by selecting clinical practice guidelines for evaluation, abstracting recommendations and disclosure statements from the guidelines and collecting information on funding sources and conflict of interest policies for the organizations producing the guidelines from their websites and by survey. The study received ethics approval from the University of Calgary Conjoint Health Research Ethics Board (REB13-0048, see S1 Text for research ethics board application).

### Selection of Guidelines

Two authors (P. C. and H. T. S.) independently reviewed all clinical practice guidelines posted to the US Agency for Healthcare Research and Quality’s National Guideline Clearinghouse website between January 1 and December 31, 2012 (accessed June 9, 2013) [14]. We included clinical practice guidelines produced by organizations whose membership and scope were national or international. We excluded clinical practice guidelines produced by organizations whose membership was restricted to allied health professionals (e.g., nursing associations) or corporations (e.g., health maintenance organizations, insurance companies, etc.); we also excluded guidelines that provided no specific recommendations (suggestions on the best course of clinical action, e.g., “We recommend…”) [15]. These criteria were used to identify clinical practice guidelines that would include recommendations that targeted clinical practices, were related to biomedical products, and could be widely disseminated. Disagreements about which guidelines met these exclusion criteria were resolved by discussion.

### Abstraction of Data from Guidelines and Websites

Two authors (P. C. and K. C.) used a standardized and pilot-tested data collection tool to abstract the data (with each author reviewing half of the clinical practice guidelines and associated websites). To evaluate the reliability of the data abstraction process, both authors independently abstracted a random sample of 10% of the guidelines and associated websites.

We abstracted data from clinical practice guidelines and the National Guideline Clearinghouse website on reported sources of funding, conflict of interest policies (or a link to a policy), and disclosures of conflicts of interest. All recommendations (suggestions on the best course of clinical action, e.g., “We suggest…”) [15] provided within each clinical practice guideline were individually classified according to whether or not they were related to a biomedical product (i.e., pharmaceutical product or medical technology product). Those recommendations classified as being related to a biomedical product were further classified as positive (recommending the product), neutral (neither recommending nor advising against use of the product), or negative (advising against use of the product) as part of a clinical action management approach using a classification scheme derived from the American College of Cardiology/American Heart Association Task Force on Practice Guidelines and GRADE [16,17]. Biomedical products were classified as patented/exclusive if the patent/exclusive expiry date for the agent was listed as 2013 or later per *Approved Drug Products with Therapeutic Equivalence Evaluations*, *33rd edition*, published by the US Department of Health and Human Services [18].

We abstracted data from the websites of the organizations producing the clinical practice guidelines on disclosure of funding from biomedical companies, solicitation of funding from biomedical companies (defined as invitation and/or instruction on how a corporation could provide funding support to the organization—website tabs/links labelled “opportunities for corporate sponsorship,” “opportunities for sponsorship at upcoming scientific meetings,” etc.), and conflict of interest policies to manage relationships between the organization and biomedical companies.

### Survey Development and Administration

The survey instrument (see S2 Text) was designed to obtain information about organizational characteristics (type of organization, membership), funding sources (annual revenue, funding sources), and conflict of interest policies (existence of a policy and specific procedures for managing conflicts of interest) for guideline production. A list of 18 procedures for managing conflicts of interest was derived from those recommended by the Institute of Medicine [3], the Council of Medical Specialty Societies [19], and the American College of Chest Physicians [20]; the survey was used to inquire which of these procedures were included in the organization’s conflict of interest policy. For organizations with a conflict of interest policy, a copy was requested. An assessment of the survey’s face validity, clarity, length, and completeness was performed using semi-structured interviews (pretesting) with physicians with experience in clinical practice guideline development prior to its distribution [21].

We searched the websites of the organizations that produced the clinical practice guidelines to identify organizational contacts. Representatives were contacted via email and telephone to identify the most appropriate person within the organization to complete the survey. The individual designated by the organization was sent an email cover letter explaining the purpose of the study and a link providing access to a secure, web-based survey (see S2 Text). Participation in the survey was voluntary; consent was inferred from survey completion. Reminders (emails at 4, 8, and 12 wk and a telephone call at 12 wk) were sent to those who did not respond [22].

### Data Analysis

Descriptive statistics (proportions and medians and interquartile ranges [IQRs]) were used to report the data abstracted from the clinical practice guidelines, organizational websites, and surveys. Data were reported for all responses to survey questions, and missing values were not imputed. Agreement on the selection of clinical practice guidelines for inclusion in the study and data abstraction from clinical practice guidelines and websites was assessed with Cohen’s kappa coefficient and Cohen’s weighted kappa coefficient [23]. We tested for associations between the reported number of procedures used by organizations for managing conflicts of interest and recommendations (number and nature) and disclosure statements provided in guidelines using Poisson and logistic regression models, respectively. To account for the interdependence of observations (organizations producing more than one guideline), we used robust estimates of variance (generalized estimating equations) [24]. Analyses were calculated using Stata (version 13.1, StataCorp).

## Results

### Selection and Review of Guidelines, Organizations, Websites, and Surveys

**Fig 1:**
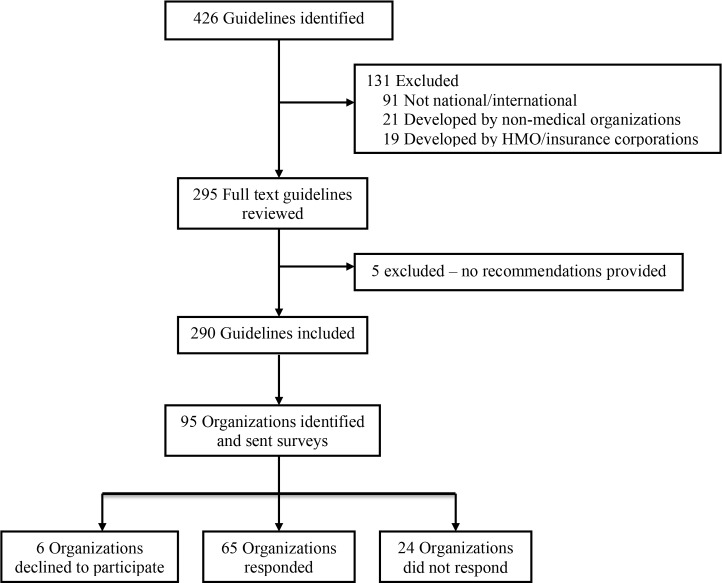
Selection of clinical practice guidelines and organizations producing the guidelines. Clinical practice guidelines produced by national/international medical organizations and posted on the National Guideline Clearinghouse website (http://www.guideline.gov/) from January 1 to December 31, 2012. HMO, health maintenance organization.

Fig 1 summarizes the selection of clinical practice guidelines and producing organizations. Our search of the National Guideline Clearinghouse identified 426 clinical practice guidelines posted between January 1 and December 31, 2012. We excluded a total of 136 clinical practice guidelines because they were produced by organizations whose membership and scope were primarily at the local or state level (*n* = 91) or whose membership was restricted to allied health professionals (*n* = 21); the guidelines were produced by health maintenance/insurance corporations (*n* = 19); or the guidelines did not provide specific recommendations (*n* = 5). We identified 290 clinical practice guidelines (see S3 Text) produced by 95 national/international medical organizations (see S4 Text) for inclusion in the study. Websites were identified for all 95 (100%) organizations (see S4 Text). The survey was sent to a representative of each organization between December 5, 2013, and April 21, 2014, of which 24 did not respond, six declined to participate, and 65 (65/95, 68%) responded/completed the survey (see S1 Data). Agreement between reviewers on inclusion of clinical practice guidelines in the study (kappa 0.973) and abstraction of data from guidelines and websites (kappa 0.802 for binary data, weighted kappa 0.997 for ordinal data) was excellent.

### Organizations Producing Clinical Practice Guidelines

The characteristics of the organizations that produced the clinical practice guidelines are summarized in Table 1; these characteristics were broadly similar across the organizations that did and did not respond to the survey. The organizations producing the clinical practice guidelines were primarily professional associations (67%) or disease/condition interest groups (21%). The self-reported yearly revenues of the organizations ranged from less than $1 million to over $50 million.

**Table 1:** Characteristics of the organizations that produced the clinical practice guidelines.

Characteristic	Organizations			*p*-Value^2^
	All (*n* = 95)	Survey Response (*n* = 65)	No Survey Response^1^ (*n* = 30)	
**Number of guidelines, median (IQR)**	2 (1–3)	2 (1–3)	1 (1–3)	0.644^3^
**Type of Organization**				0.092
Professional association^4^	64 (67%)	48 (74%)	16 (53%)	
Disease/condition interest group^5^	20 (21%)	12 (18%)	8 (27%)	
Governmental organization^6^	11 (12%)	5 (8%)	6 (20%)	
**Number of reported members, median (IQR)** ^**7**^	6,800 (1,600–26,000)	6,800 (1,600–26,000)	—	—
**Clinical focus** ^**8**^				0.131
Medical	78 (82%)	52 (80%)	26 (87%)	
Surgical	32 (33%)	26 (40%)	6 (20%)	
**Membership**				0.015
National	72 (76%)	54 (83%)	18 (60%)	
International	23 (24%)	11 (17%)	12 (40%)	
**Specific patient demographic population**				0.919^9^
Pediatric	5 (5%)	4 (6%)	1 (3%)	
Female	5 (5%)	3 (5%)	2 (7%)	
Geriatric	1 (1%)	1 (1%)	0 (0%)	
**Total annual revenue reported** ^**10**^				
Less than $1 million	2 (5%)	2 (5%)	—	—
$1 million to $10 million	13 (32%)	13 (32%)	—	—
$11 million to $50 million	13 (32%)	13 (32%)	—	—
More than $50 million	13 (32%)	13 (32%)	—	—

Data presented as number (percentage) unless otherwise indicated.

^1^Did not respond or declined to participate.

^2^Pearson chi-squared test unless otherwise specified.

^3^Kruskal–Wallis equality-of-populations rank test.

^4^For example, the American College of Rheumatology.

^5^For example, World Federation of Hemophilia.

^6^For example, United States Preventive Services Task Force.

^7^Among the 60 organizations that reported membership in the survey responses.

^8^Numbers add to greater than 100% as some organizations had both a medical and surgical focus.

^9^Fischer’s exact test.

^10^Among the 41 organizations reporting yearly revenue in survey responses. Currency was not specified in the survey; responses are assumed to be in US dollars.

#### Financial relationships

Sixty-three percent (60/95) of organizations reported receiving funds from biomedical companies, as identified from their website (52/95, 55%) and/or reported in the survey response (20/95, 21%). Sixty-four organizations (64/95, 67%) solicited funds from biomedical companies on their website. Among the 38 organizations that responded to the survey question inquiring about funding sources, organizations reported receiving funds from government sources (9/38, 24%), organizational activities such as membership dues (33/38, 87%), charitable donations (15/38, 39%), donations from not-for-profit companies (12/38, 32%), and donations from for-profit companies (20/38, 53%).

#### Policies for managing conflicts of interest

**Fig 2:**
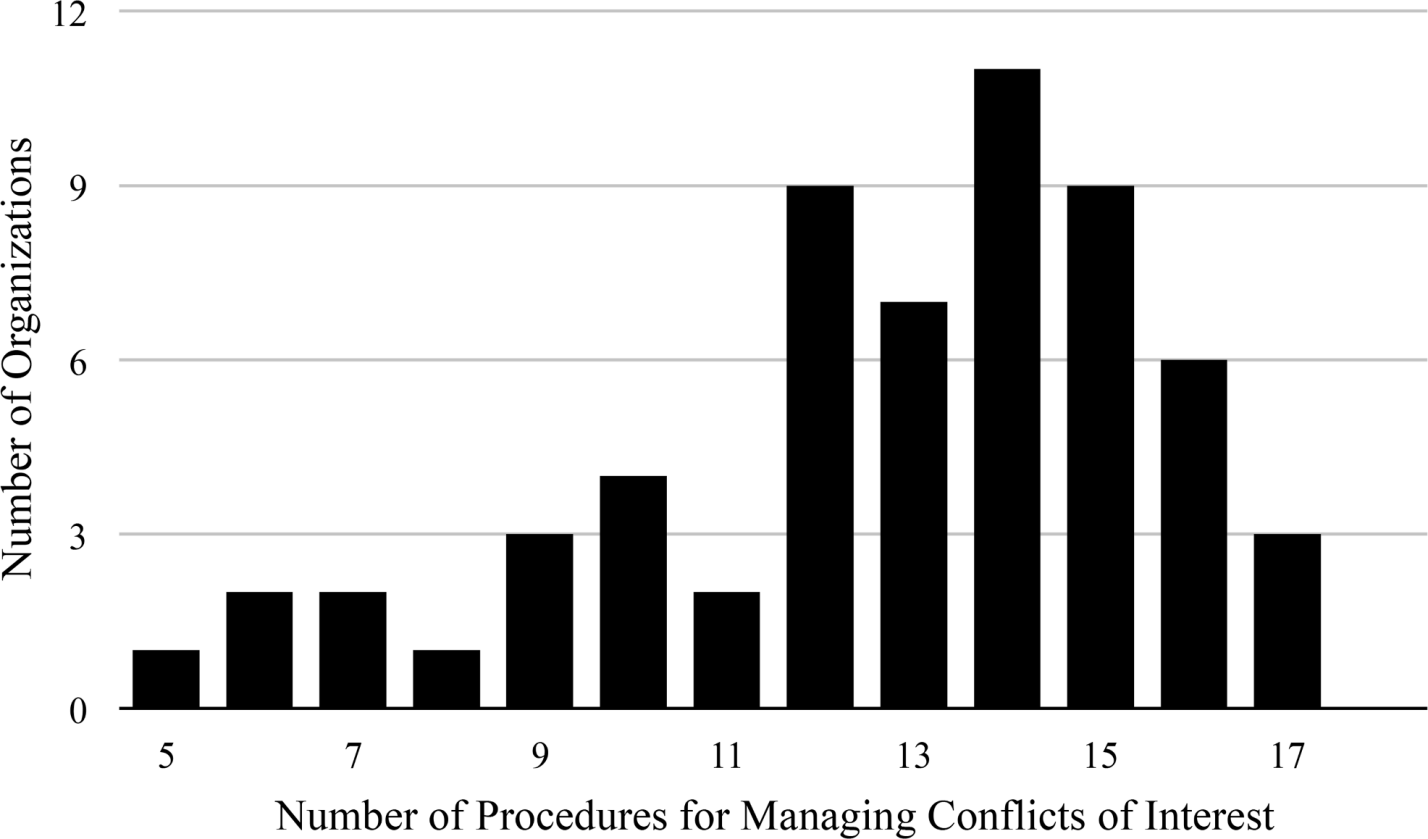
Number of procedures for managing conflicts of interest reported by organizations producing clinical practice guidelines. Number of conflict of interest procedures recommended by the Institute of Medicine, the Council of Medical Specialty Societies, and the American College of Chest Physicians (*n* = 18) reported to be used by organizations producing clinical practice guidelines in survey responses (*n* = 60).

The majority of organizations reported having a policy for managing conflicts of interest (76/95, 80%), as identified from their website (66/95, 69%) or reported in the survey response (55/95, 58%). Among the 69 conflict of interest policies available for review (either provided by organizations or available from their websites), 31 (31/69, 45%) made specific reference to practices related to guideline development. Fig 2 displays the number of specific procedures for managing conflicts of interest that were reported by organizations in the survey responses as being in their conflict of interest policies (*n* = 60). A description of the procedures is provided in Table 2. The majority of organizations required that committee members disclose financial conflicts of interest (59/60, 98%), that conflicts be reviewed prior to clinical practice guideline production (54/60, 90%), and that the majority of committee members be free of conflict (41/60, 68%). Most organizations reported publishing committee member conflicts of interest within the clinical practice guidelines (55/60, 92%). Most organizations did not permit industry partners to directly fund clinical practice guideline development (47/60, 78%), participate in selection of committee members (49/60, 82%), or review the clinical practice guidelines prior to release (40/60, 67%). A minority of organizations had a standing committee to oversee organizational conflicts of interest (24/60, 40%) or procedures in place to manage violations of the conflict of interest policy (15/60, 25%).

**Table 2:** Procedures for managing conflicts of interest reported by organizations producing clinical practice guidelines.

Procedures for Managing Conflicts of Interest^1^	Number (Percent) of Organizations (*n* = 60)^2^
**Disclosure requirements for committee members**	
Financial conflicts of interest	59 (98%)
All financial conflicts of interest regardless of perceived relevance to the guideline	49 (82%)
Academic/intellectual conflicts of interest	44 (73%)
**Composition of the guideline committee**	
Attempt to recruit committee members without conflicts of interest	42 (70%)
Majority of committee members must be free of conflicts of interest	41 (68%)^3^
Committee chair must be free of conflicts of interest	39 (65%)
Standing committee within the organization oversees institutional conflicts of interest	24 (40%)
**Management of conflicts of interest during guideline development**	
Conflicts of interest are reviewed prior to guideline production	54 (90%)
Committee members with conflicts of interest do not deliberate, draft, or vote on related recommendations	36 (60%)
Committee members are blinded to which companies financially contributed to guideline production	6 (10%)
**Conflict of interest disclosure and guideline publication**	
Committee member conflicts of interest are published in guidelines	55 (92%)^4^
Guidelines peer reviewed by clinicians not involved in production of the guideline	54 (90%)
Guidelines subject to independent review by the journal in which they are published	35 (58%)
**Role of industry partners in guideline development**	
Industry partners not permitted to directly fund clinical guideline development	47 (78%)^5^
Industry partners do not participate in the selection of clinical guideline committee members	49 (82%)
Industry products referred to in the clinical guidelines by their generic name	43 (72%)
Industry partners not permitted to review clinical guidelines prior to release	40 (67%)
**Breakdown of conflict of interest policy**	
Procedure for managing breakdown/violation of conflict of interest policy	15 (25%)

^1^Eighteen procedures for managing conflicts of interest derived from published recommendations by the Institute of Medicine, the Council of Medical Specialty Societies, and the American College of Chest Physicians.

^2^Data presented as number (percent) among the 60 organizations reporting specific procedures for managing conflicts of interest in survey responses.

^3^Among these organizations, 25 (61%) published a guideline during the study period that disclosed a majority of committee members to have financial relationships with biomedical companies.

^4^Among these organizations, nine (16%) published a guideline during the study period that did not include a committee member disclosure statement.

^5^Among these organizations, three (6%) published a guideline during the study period that disclosed direct funding/support from a biomedical company.

### Clinical Practice Guidelines

The characteristics of the clinical practice guidelines are summarized in Table 3. The majority of clinical practice guidelines were produced in the United States (65%) and were focused on internal medicine and its subspecialties (42%). The clinical practice guidelines included a total of 4,057 guideline committee members, with a median of 13 members per guideline (IQR 8–17). The median number of recommendations per clinical practice guideline was 9.5 (IQR 4–24), and these recommendations included recommendations regarding pharmaceutical products and medical technologies. Sixty-two percent of clinical practice guidelines had been published in peer-reviewed journals at the time of data collection.

**Table 3:** Characteristics of the clinical practice guidelines.

Characteristics	Guidelines (*n* = 290)
**Medical specialty**	
Internal medicine and subspecialties	121 (42%)
Radiology	67 (23%)
Obstetrics and gynecology	23 (8%)
Neurology	21 (7%)
Urology	15 (5%)
Family practice	15 (5%)
Other	28 (10%)
**Country of origin**	
United States	189 (65%)
United Kingdom	54 (19%)
Other	47 (16%)
**Number of guideline committee members per guideline, median (IQR)**	13 (8–17)
**Number of recommendations per guideline, median (IQR)**	9.5 (4–24)
**Included in recommendations**	
Biomedical products	167 (58%)
Pharmaceutical products	165 (57%)
Medical technology products	13 (4%)
Products under patent	60 (21%)
**Published in a peer-reviewed journal**	158 (62%)

Data presented as number (percentage) unless otherwise indicated.

#### Disclosure statements

Table 4 summarizes the types of disclosures found in the disclosure statements contained within the clinical practice guidelines. The majority of clinical practice guidelines (188/290, 65%) included disclosure statements regarding direct funding and support provided for clinical practice guideline development. A minority of clinical practice guidelines disclosed that direct funding/support from biomedical companies was either received (18/290, 6%) or not received (90/290, 31%). A majority of clinical practice guidelines (165/290, 57%) disclosed receipt of direct funding/support from the organization producing the guideline.

**Table 4:** Disclosure statements contained within clinical practice guidelines.

Type of Disclosure	Guidelines (*n* = 290)
**Direct funding/support for guideline development** ^1^	
No disclosure statement	102 (35%)
Disclosed that no funding/support was received	17 (6%)
Disclosed that no funding/support from the biomedical industry was received	90 (31%)
Disclosed receipt of funding/support from biomedical industry	18 (6%)
Disclosed receipt of funding/support from organization producing guideline	165 (57%)
Disclosed receipt of funding/support from third party organization not directly involved in producing the guideline^2^	4 (1%)
**Financial relationships of guideline committee members** ^3^	
No disclosure statement	143 (49%)
Disclosed absence of financial relationships	30 (10%)
Disclosed presence of financial relationships	117 (40%)
Disclosed number of guideline committee members with a financial relationship	773 (38%)^4^
Number of financial relationships disclosed per guideline committee member^5^, mean	4.8
**Financial relationships of the organization producing the guideline** ^3^	
No disclosure statement	286 (99%)
Disclosed absence of financial relationships	0 (0%)
Disclosed presence of financial relationships	4 (1%)

Data presented as number (percentage) unless otherwise indicated.

^1^Types of disclosure sum to greater than 290 (100%) as more than one disclosure was provided in some guidelines.

^2^Funding/support disclosed from the Light of Life Foundation, the Thyroid Cancer Survivors’ Association, the National Institutes of Health Office of AIDS Research, the National Institute of Diabetes and Digestive and Kidney Diseases, and the Centers for Disease Control and Prevention.

^3^Financial relationships with biomedical companies.

^4^Denominator is the 2,040 committee members of guidelines that provided disclosure statements.

^5^Among guideline committee members disclosing a financial relationship.

Disclosure statements for committee member financial relationships were provided in half of the clinical practice guidelines (147/290, 51%). A small minority of clinical practice guidelines disclosed the absence of financial relationships between committee members and biomedical companies (30/290, 10%).

Four clinical practice guidelines (4/290, 1%) provided financial relationship disclosure statements for the organization producing the guideline. The remaining clinical practice guidelines (286/290, 99%) did not provide a disclosure statement regarding the organization’s financial relationships.

### Relationship between Organizations’ Conflict of Interest Policies and Guideline Recommendations and Disclosures

The majority (55/60, 92%) of organizations that reported procedures for managing conflicts of interest indicated that they had a policy specifically for managing conflicts of interest during guideline development. A minority (50/290, 17%) of clinical practice guidelines made reference to a conflict of interest policy of the producing organization. Three of the procedures reported by organizations for managing conflicts of interest (Table 2) could be compared to the disclosures provided in the guidelines produced by the organizations during the study period (Table 4). First, among organizations that reported that committee member conflicts of interest were published in guidelines, nine (9/55, 16%) produced a guideline that did not include a committee member disclosure statement. Second, among organizations that reported that the majority of committee members must be free of conflicts of interest, over half (25/41, 61%) produced a guideline that disclosed financial relationships with biomedical companies for a majority of the committee members. Third, among the organizations that reported that industry partners were not permitted to directly fund clinical practice guideline development, three (3/47, 6%) produced a guideline that disclosed direct funding/support from a biomedical company.

Table 5 summarizes recommendations and disclosures provided in clinical practice guidelines (*n* = 158) according to the number of procedures for managing conflicts of interest reported by the producing organization (*n* = 60). There was an association between the number of conflict of interest procedures reported by an organization (18 potential items, response range 5–17) and the number of guidelines produced (rate ratio [RR] 1.10, 95% CI 1.03–1.17, *p* = 0.003). Organizations with more comprehensive policies (per additional procedure) produced guidelines that included more recommendations regarding biomedical products (RR 1.05, 95% CI 1.03–1.07) but fewer recommendations regarding patented biomedical products (RR 0.94, 95% CI 0.90–0.98). Clinical practice guidelines produced by organizations reporting more comprehensive conflict of interest policies included fewer positive (RR 0.91, 95% CI 0.86–0.95) and more negative (RR 1.32, 95% CI 1.09–1.60) recommendations regarding patented biomedical products. These clinical practice guidelines were more likely to include disclosure statements for direct funding sources (odds ratio [OR] 1.31, 95% CI 1.10–1.56) and for financial relationships of guideline committee members (OR 1.36, 95% CI 1.09–1.79) but not for financial relationships of the organization (zero disclosures).

**Table 5:** Clinical practice guideline recommendations and disclosures according to the number of procedures in an organization’s conflict of interest policy.

Recommendation or Disclosure	Number per Guideline^1^	RR or OR (95% CI)^2^	*p*-Value
**All recommendations**	33.25	1.04 (1.03, 1.05)	<0.001
**Recommendations regarding biomedical products**	12.17	1.05 (1.03, 1.07)	<0.001
Positive recommendations	8.75	1.00 (0.98, 1.02)	0.696
Neutral recommendations	1.28	0.96 (0.92, 1.01)	0.114
Negative recommendations	2.13	1.01 (0.97, 1.05)	0.662
**Recommendations regarding patented biomedical products**	1.37	0.94 (0.90, 0.98)	0.003
Positive recommendations	0.97	0.91 (0.86, 0.95)	<0.001
Neutral recommendations	0.2	0.90 (0.81, 1.00)	0.061
Negative recommendations	0.2	1.32 (1.09, 1.60)	0.004
**Disclosures**			
Direct funding/support disclosure statement	0.55	1.31 (1.10, 1.56)	0.003
Committee disclosure statement	0.76	1.36 (1.09, 1.79)	0.006
Organization disclosure statement	0	—	—

These data are for the 60 organizations producing 158 clinical practice guidelines that self-reported procedures included in their conflict of interest policies.

^1^Mean number of recommendations or disclosures per guideline.

^2^RR for recommendations and OR for disclosures for each additional procedure for managing conflicts of interest derived from published recommendations by the Institute of Medicine, the Council of Medical Specialty Societies, and the American College of Chest Physicians.

## Discussion

Our study described financial relationships between organizations that produce clinical practice guidelines and the biomedical industry, and the policies employed to manage conflicts of interest. The results demonstrated that the majority of organizations reported financial relationships with biomedical companies. Most organizations had policies and procedures to manage conflicts of interest; however, there was variation in the procedures included within the policies, and a minority of policies specifically considered production of clinical practice guidelines. Two-thirds of clinical practice guidelines provided disclosure statements for direct funding sources for the guideline, and half provided disclosure statements for guideline committee members. Only 1% of clinical practice guidelines provided disclosure statements for the organizations producing the guidelines.

A growing body of research over the past two decades has described and explored the implications of financial relationships between the medical community and biomedical companies [5,25–28]. For example, a systematic review by Licurse et al. reported that patients, research participants, and readers of medical journals perceive financial relationships between physicians and biomedical companies as impacting the quality and cost of healthcare and believe that these relationships should be disclosed [29]. Journals [30,31], professional societies [19,20,32], government agencies [33], and the biomedical industry [34] have implemented strategies for managing conflicts of interest. A systematic review by Norris et al. in 2011 reported that financial relationships between clinical practice guideline committee members and biomedical companies are common [4] but may be increasingly disclosed [2]. Our study adds to this body of literature by suggesting three key issues that should be considered to further improve the prevention and management of conflicts of interest in the financial relationships between organizations that produce clinical practice guidelines and the biomedical industry.

First, organizations that produce clinical practice guidelines should develop conflict of interest policies to manage relationships with biomedical companies, should ensure these policies address the production of guidelines, and should make the policies available to guideline users. In our study, approximately one in five organizations did not have a conflict of interest policy, and less than half of policies specifically addressed the production of guidelines. Furthermore, conflict of interest policies were infrequently referenced in the guideline text, leaving readers with uncertainty about how conflicts were prevented or managed. The content of the conflict of interest policy is essential for critical appraisal [35], and policies should be made publicly available. In 2009, the Institute of Medicine recommended that journals and websites that publish clinical practice guidelines require organizations to “describe (or provide an Internet link to) the developer’s conflict of interest policy” [3]. Although it is unclear what an optimal conflict of interest policy should include, the data abstraction elements used in the present study—which were derived from the recommendations provided by the Institute of Medicine [3], the Council of Medical Specialty Societies [19], and the American College of Chest Physicians [20]—could serve as a starting template.

Second, disclosure of financial relationships is necessary for transparency and managing conflicts of interest [36,37]. A clinical practice guideline can be funded directly, whereby an entity provides funds for production of a particular guideline, or indirectly, whereby an entity provides funds to an organization that produces clinical practice guidelines, and these funds are then applied to the organization’s programs including guideline production. Both forms of funding represent conflicts of interest. The Institute of Medicine recommends that organizations disclose “sources and amounts of indirect or direct funding received for development of the guideline” [3]. Our study suggests that while disclosure statements regarding direct funding and support for committee members are increasingly provided in clinical practice guidelines [2,4,38,39], disclosure of financial relationships between the organizations that produce guidelines and biomedical companies is uncommon.

Third, mechanisms are needed to manage breakdowns in policies to manage conflicts of interest. Financial relationships between organizations that produce clinical practice guidelines and the biomedical industry are common and likely complex. We anticipate that even when organizations have policies to manage conflicts of interest, breakdowns in execution of policies are inevitable. For example, among the organizations that responded to the survey, 68% indicated that their policies stipulated that the majority of committee members must be free of conflicts of interest, yet, of these, 61% published a guideline included in our study for which the majority of committee members disclosed financial relationships with biomedical companies. Similarly, although 92% of organizations responded in the survey that they published committee member disclosure statements within their guidelines, 18% of these organizations published at least one guideline included in our study that provided no committee member disclosure statement. These discrepancies reflect the complexity of managing conflicts of interest related to producing a clinical practice guideline. A solution advocated by the Institute of Medicine is that organizations create a standing committee to oversee organizational conflicts of interest as well as procedures to manage violations of the conflict of interest policy [3]. A minority of organizations in our study reported using either of these approaches.

The results of our study need to be interpreted within the context of the study’s limitations. First, we sampled a single source of clinical practice guidelines, the National Guideline Clearinghouse, although this is the largest repository of clinical practice guidelines. Furthermore, only English language guidelines are included on the clearinghouse website, and we restricted our focus to guidelines produced by national/international medical organizations, which may limit the transferability of our results. Second, data abstraction was performed by one reviewer, which could introduce error or bias. To guard against this risk, data abstraction was sequentially performed for clinical practice guidelines and websites prior to survey administration, and independent blinded abstraction of a random sample of 10% of the clinical practice guidelines and websites by a second reviewer demonstrated excellent reliability. Third, we developed a parsimonious survey instrument to encourage participation, but this limited data collection. For example, our study provides no data on how organizations managed conflicts of interest for their staff members. Finally, we depended on organizations’ self-report through survey responses and website postings. This limits our ability to fully describe relationships between organizations producing clinical practice guidelines and biomedical companies, and suggests that our analyses likely underestimate the number of these relationships.

### Conclusion

Financial relationships between organizations that produce clinical practice guidelines and the biomedical industry appear to be common. These relationships are important because they may influence, through guideline usage, the practice of large numbers of healthcare providers. We believe that to effectively manage conflicts of interest, organizations that produce clinical practice guidelines need to develop robust conflict of interest policies that include procedures for managing violations of the policy, make the policies publicly available, and disclose all financial relationships with biomedical companies.

## Supporting Information

S1 Data Survey data. (XLSX)

S1 Text Ethics application. (PDF)

S2 Text Survey instrument. (PDF)

S3 Text Clinical practice guidelines bibliography. (DOCX)

S4 Text Organizations producing clinical practice guidelines. (DOCX)

S1 STROBE Checklist of items that should be included in reports of cross-sectional studies. (DOC)

## Abbreviations

IQR: interquartile range
OR: odds ratio
RR: rate ratio
