# Supplementary material for: Financial Relationships between Organizations That Produce Clinical Practice Guidelines and the Biomedical Industry: A Cross-Sectional Study
Source: PLoS Med. 2016 May 31;13(5):e1002029. doi: 10.1371/journal.pmed.1002029 (PMC4887051; doi:10.1371/journal.pmed.1002029)
Supplement: S1 Text — (PDF) [file pmed.1002029.s002.pdf]

# A Cross-Sectional Study of Financial Relationships Between Organizations that Produce Clinical Practice Guidelines and the Biomedical Industry

*Paul Campsall , Kate Colizza, Sharon Straus, Henry Thomas Stelfox*

Conjoint Health Research Ethics Board
Research Services Office
Energy Resources Research Building (ERRB)
Suite N140, 3512-33 Street NW
Calgary, Alberta, T2L 2A6
Telephone: (403) 220-7990
Fax: (403) 289-0693
chreb@ucalgary.ca

July 25, 2013

Thomas Stelfox

Critical Care Medicine

**Dear Thomas Stelfox :**

**RE: Conflict of interest in clinical practice guidelines**

**Ethics ID: REB13-0048**

The above-named research, including protocol for ethics.pdf, V.1, May 22, 2013; ethics intro letter.pdf, V.1, May 22, 2013; survey.pdf, V.1, May 22, 2013, has been granted ethical approval by the Conjoint Health Research Ethics Board of the Faculties of Medicine, Nursing and Kinesiology, University of Calgary, and the Affiliated Teaching Institutions. The Board conforms to the Tri-Council Guidelines, ICH Guidelines and amendments to regulations of the Food and Drugs Act re clinical trials, including membership and requirements for a quorum.

You and your co-investigators are not members of the CHREB and did not participate in review or voting on this study.

Please note that this approval is subject to the following conditions:

1. A renewal must be submitted by **July 25, 2014** , containing the following information:
   i. The number of subjects recruited;
   ii. A description of any protocol modification;
   iii. Any unusual and/or severe complications, adverse events or unanticipated problems involving risks to subjects or others, withdrawal of subjects from the research, or complaints about the research;
   iv. A summary of any recent literature, finding, or other relevant information, especially information about risks associated with the research;
   v. A copy of the current informed consent form;
   vi. The expected date of termination of this project.
2. A Final Report must be submitted at the termination of the project.

Please accept the Board's best wishes for success in your research.

| **Sincerely,** | **Date:** |
|---|---|
| David Richard Leslie Cawthorpe, Acting Chair , CHREB | August 2, 2013 |

IRISS

Date: January 11, 2016 at 12:26:08 PM MST

Print Close

# Research Ethics Board

If your study involves disclosure of personal health information you are required to apply to either CHREB or HREBA. For University of Calgary applications, the Board of record is determined by the faculty or affiliation of the PI.

*All questions preceded by a red asterisk (*) are required responses that map you to the application sections that are relevant to your study. All questions within the section to which you are mapped must be addressed.*

1.0 * **Select the appropriate Research Ethics Board:**
CHREB

# Getting Started – Study Staff, Funding, Location

By completing the information on the first page and clicking either **'Save'** or **'Continue'** you will create a new REB Certification. You will be able to access this application through your Personal Page. As you complete this application form, you can save your work, exit and come back to it at any time.

The people named on this first page constitute the team's Key Personnel and will have the ability to access the application and assist in its completion. However, only the Principal Investigator will have the ability to submit this application when complete.

**Note:** by listing people on this first page, you will be providing them with the ability to see limited information about your personal contact information, your training and experience, and project funding information.

*All questions preceded by a red asterisk (*) are required responses that map you to the application sections that are relevant to your study. All questions within the section to which you are mapped* ***must be addressed***.

**BE AWARE** that while some navigation features in this form will prompt you to save, if you click the **'Back'** button either in this form or in your web-browser before saving your work, you will lose your information without any warning. Save your work often!

**Study Identification**

1.0 * **Purpose of Application:**
New application

2.0 * **Short Study Title:** ***(250 characters)***
Conflict of interest in clinical practice guidelines

3.0 * **Long Study Title:**
Conflict of interest in clinical practice guidelines

4.0 * **Name of Principal Investigator:**
Thomas Stelfox

5.0 **Co-Investigators: can edit the application but do not receive system notifications:**

| Profile | Last | First | Dept | E-Mail | Phone | Cell |
|---|---|---|---|---|---|---|

There are no items to display

**Study Team: Student, Medical Resident, Post Doctoral Fellow Co-Investigators, Co-Investigator: can edit the application and will receive all system notifications for this study:**

| Profile | Last | First | Dept | E-Mail | Phone | Cell | Status |
|---|---|---|---|---|---|---|---|
| 00008563 | Campsall | Paul | | paulcampsall@gmail.com | 403-875-8912 | | University Student |

6.0 **Study Coordinator(s), Research Nurse or Research Assistant(s): can edit the application and will receive all system notifications for this study:**

| Last | First | E-Mail | Phone | Cell |
|---|---|---|---|---|
| Boyd | Jamie | jamboyd@ucalgary.ca | 403-210-7490 | |

7.0 **External Collaborator(s): List any other people who need access to information contained in this study:**

| | Last Name | First Name | Phone Number | Email | Role on Study | Other Role on Study |
|---|---|---|---|---|---|---|
| View | Straus | Sharon | | sharon.straus@utoronto.ca | Co-investigator | |

## Application Type

*All questions preceded by a red asterisk (*) are required responses that map you to the application sections that are relevant to your study. All questions within the section to which you are mapped must be addressed.*

1.0 * **Select the type of study:**
Medical Student, Resident

## Funding Information

*All questions preceded by a red asterisk (*) are required responses that map you to the application sections that are relevant to your study. All questions within the section to which you are mapped must be addressed.*

1.0 * **Proposed research is:**
Unfunded

2.0 **Specify funding source:**

3.0 **University of Calgary funding categories:**

# Conflict of Interest

*All questions preceded by a red asterisk (*) are required responses that map you to the application sections that are relevant to your study. All questions within the section to which you are mapped must be addressed.*

**1.0** *** Are any of the investigators or their immediate families receiving any personal remuneration from the funding of this study that is not accounted for in the study budget?**
no

**If Yes, explain:**

**2.0** *** Do any of the investigators or their immediate families have any proprietary interests in the product under study or the outcome of the research including patents, trademarks, copyrights, and licensing agreements?**
○ Yes ● **No**

**If Yes, explain:**

**3.0** **Is there any compensation for this study that is affected by the study outcome?**
○ Yes ● **No**

**If Yes, explain:**

**4.0** **Do any of the investigators or their immediate families have equity interest in the sponsoring company?**
○ Yes ● **No**

**If Yes, explain:**

**5.0** **Do any of the investigators or their immediate families receive payments of other sorts, from the sponsor?**
○ Yes ● **No**

**If Yes, explain:**

**6.0** **Are any of the investigators or their immediate family, members of the sponsor's Board of Directors, Scientific Advisory Panel or comparable body?**
○ Yes ● **No**

**If Yes, explain:**

**7.0** **Do you have any other relationship, financial or non-financial, that, if not disclosed, could be construed as a conflict of interest?**
○ Yes ● **No**

**If Yes, explain:**

## Impact and Operational Approvals

Some research may require operational approval and/or approval by another REB depending on the nature and location of the research.

**Research impacting AHS** (i.e., use of facilities, staff, patients)
For research undertaken by AHS staff or using AHS resources, appropriate authorizations (i.e., signatures) must be obtained from those areas where operations will be impacted by the protocol. This is to ensure that the individual responsible for the area is aware of, and able to, support your study. Complete and upload the Operational/Department Approval Form in the Document section 'Other Documents' at the end of the application.

**Research impacting the University of Calgary** (i.e., use of facilities, staff, students)
For research undertaken by University of Calgary faculty, staff or students, or using University of Calgary resources, approval must be obtained from the PI's Department Head. Complete and upload the Operational/Department Approval Form in the Document section 'Other Documents' at the end of the application.

**Research impacting the community** (i.e., use of staff, facilities or members of public agencies, private organizations)
For research undertaken in the community, submit confirmation of the agency's support of the research undertaking (e.g., email, letter of support - Document section 'Other Documents').

**PI attestation:**
As the PI, you are assuming responsibility for the ethical conduct of the study. This includes ensuring respect for human research participants through adherence to local, provincial and national research standards (e.g., TCPS 2) and relevant legislation (e.g., Health Information Act, Hospitals Act). You are required to provide your signature on the Operational/Departmental Approval form, as an indication that you have accepted these responsibilities.

*All questions preceded by a red asterisk (*) are required responses that map you to the application sections that are relevant to your study. All questions within the section to which you are mapped must be addressed.*

**1.0** * **List the locations where the research will be undertaken:**
Research will be computer based, and will be done within the city of Calgary.

**2.0** * **Indicate if the study team will utilize or access facilities, programs, resources, staff, students, specimens, patients or their records, at any of the sites affiliated with the following: *(select all that apply)***

Not Applicable

**List all departments and/or facilities:**

Note: if you intend to use the resources of AHS or Covenant Health for your research, limited sections of this application will be made accessible to a designate of the organization so they can review and approve the use of resources. AHS and Covenant Health will receive ongoing notice of the study's ethics certification status.

**3.0** * **Indicate if the proposed research requires ethics approval from other Research Ethics Board(s):** ***(Select all that apply)***

Not applicable

**If Other, list the REB:**

Name

There are no items to display

**4.0** **If this application is closely linked to research previously approved by an REB, provide the Ethics ID Number, REB name or other identifying information. Upload the approval letter in the Documentation Section ("Other Documents") at the end of the application.**

**5.0** **Has this study undergone independent, scientific review (this includes review by a funding body, internal peer review, approval/certification by a Canadian REB, or for graduate student work, supervisory committee sign-off)?**

◯Yes ◯No

**Note:** If Yes, upload the review, certificate or notice of award in the Documentation section 'Other Documents' at the end of this application.

**Archived Question:**

If the proposed research is above minimal risk and is not funded via a competitive peer review grant or industry-sponsored clinical trial, the REB will require evidence of scientific review. Provide information about the review process and its results if appropriate:

**Archived Response:**

Minimal risk survey based study.

## Study Objectives and Design

The REB's main interest here is what the research will be doing with participants and he/she undertakes the study so that they can assess potential risks to the participants and how the researcher is handling them.

All acronyms must be written out in full the first time that they appear in the application form, recruiting materials and consent materials.

*All questions preceded by a red asterisk (*) are required responses that map you to the application sections that are relevant to your study. All questions within the section to which you are mapped must be addressed.*

**1.0** **Anticipated date that research will begin:**

**2.0** **Anticipated date that research will end:**

**3.0** **Anticipated date that interaction with participants will begin:**

5/20/2013

**4.0** **Anticipated date that interaction with participants will end:**

8/19/2013

**5.0** * **__Briefly__ describe the (1) Background & Rationale, (2) Research Question & Objectives and (3) Methods:** ***(maximum of 750 words)***

Clinical practice guidelines (CPG) can have a high impact and potentially alter the practice of a large number of health care providers including physicians. In recent years there has been increased scrutiny of the potential conflicts of interest (COI) of CPG authors. A recent systematic review identified that of CPG authors, 56-87% had a potential COI. Producers of CPGs such as professional medical associations have responded to this concern with many requiring disclosure statements from authors to be published in the guidelines.

Creation of CPGs is resource intensive and often requires substantial financial input. Funding sources include governmental organizations, professional association general funds, and pharmaceutical company donations. The potential COI related to guideline producer funding has led the Institute of Medicine Committee on Conflict of Interest in Medical Research, Education, and Practice to conclude that "the risk of undue industry influence on clinical practice guidelines is significant." The committee goes on to recommend that all sources of funding, both direct and indirect, be "publicly disclose[d] with each guideline." Many guideline producers have formalized protocols to minimize potential COI. However, it is unclear to what extent COI protocols are used by guideline developers and whether pharmaceutical funding either direct or indirect is disclosed in the CPGs. To date there has not been a systematic analysis of the funding sources of CPG producers and their disclosure. Nor has there been an analysis of the procedures in place to minimize potential COI in guideline development. Therefore we propose to perform an observational study to document pharmaceutical funding of CPG producers, protocols used by CPG producers to manage potential COIs and disclosure of funding sources (direct and indirect) for guideline producers in published CPG.

Study Questions:

1) Do organizations that produce CPGs receive funding from for profit companies that sell pharmaceutical or medical technology products?

2) Do organizations that produce CPGs have operating procedures to manage potential COI during clinical guideline development?

3)Do organizations that produce CPGs disclose within the guideline their funding sources and the procedures for managing potential COI?

4) Is there an association between the strength of recommendations for a pharmaceutical product and the rigor of the procedures to manage/prevent potential COI?

5)Is there an association between the rigor of the guidelines producer's procedures to manage/prevent potential COI and the quality of the guidelines as defined by the AGREE guidelines assessment tool.

6)Is there an association between the financial COI of individual authors and the funding sources of CPG producers?

Methods:

Study Population: All guidelines entered in the National Guidelines Clearinghouse from Jan. 1st, 2012 to Dec. 31st, 2012.

Study Measures:

Each guideline will be analyzed for four items:

The sources of funding for the guideline producer.

The existence and basic elements of a procedure to minimize potential COI for the production of CPGs.

Presence of a disclosure statement within the guideline of funding sources and the procedures for managing potential COI.

The financial COI of the individual guideline authors.

Wherever this information is not available within the guideline text or guideline producer's website, an email survey will be sent to the guideline producer requesting the information.

Conclusion:

Given the potential for far-reaching impact of guidelines on clinical practice, it is essential that these publications are free of significant COI. Above the level of individual authors, financial COI in the form of funding for CPG producers may introduce significant bias; however, this issue is poorly established. A systematic analysis of potential COI related to funding at the guideline producer level is a key first step in defining the issue. Depending on the results of the analysis, there may be justification for an organized and uniform approach to minimizing and disclosing COI of this kind to allow end-users to reliably appraise clinical practice guidelines.

**6.0 Describe procedures, treatment, or activities that are above or in addition to standard practices:**

N/A

**Archived Responses:**

**Provide a lay summary of the proposed research suitable for the general public: *(maximum of 250 words)***

*Clinical practice guidelines provide recommendations to healthcare providers on how to investigate and treat a given condition. A single guideline may have a large impact and alter many doctors’ practice. There is a concern that if a pharmaceutical company were able to influence the content of the guidelines it may serve to benefit the company rather than guide best patient care. In order to minimize this potential conflict on interest, guideline authors are often required to declare any financial ties with pharmaceutical companies.*
*Guideline production is resource intensive and often guideline producers such as professional associations receive money from pharmaceutical companies that either directly or indirectly fund the creation of guidelines. To date, there has been no research study that has defined how common it is for guideline producers to receive pharmaceutical funding, and how these organizations manage this potential conflict of interest.*
*We are proposing to study potential conflicts of interest in guidelines production by examining all guidelines published in 2012 in the National Guidelines Clearinghouse (the major online repository for clinical guidelines). For each guideline we will determine the following:*
*If there was any direct or indirect pharmaceutical funding and was that funding disclosed in the guideline text*
*Does the guideline producer have a procedure in place to manage potential conflicts of interest*
*Do the guideline authors have any financial conflicts of interest and are they similar to those of the guidelines producers*

*Given the potential for far-reaching impact of guidelines on the care of patients, it is essential that these publications are free of significant financial conflicts of interest. A systematic analysis of potential COI related to funding at the guideline producer level is a key first step in addressing this issue.*

## Risk Assessment

*All questions preceded by a red asterisk (*) are required responses that map you to the application sections that are relevant to your study. All questions within the section to which you are mapped must be addressed.*

**1.0**

**Potential Physical Risks and Discomforts**

| | |
|---|---|
| * **Participants might feel physical fatigue** | No |
| * **Participants might feel physical stress, e.g. cardiovascular stress tests.** | No |
| * **Participants might sustain injury, infection, and intervention side-effects or complications.** | No |

**Archived Response:**

| | |
|---|---|
| The physical risks will be greater than those encountered by the participants in everyday life. | No |

**Potential Psychological, Emotional, Social and other Risks and Discomforts**

| | |
|---|---|
| * **Participants might feel psychologically or emotionally stressed, demeaned, embarrassed, worried, anxious, scared or distressed** | No |
| * **Participants might feel psychological or mental fatigue, e.g. intense concentration required.** | No |
| * **Participants might experience cultural or social risk, e.g. loss of privacy or status or damage to reputation.** | No |
| * **Participants might be exposed to economic or legal risk, e.g. non-anonymized workplace surveys.** | No |

**Archived Response:**

| | |
|---|---|
| The risks will be greater than those encountered by the participants in everyday life. | No |

**2.0** * **Will the risks be greater than those encountered by the participants in everyday life?**

No

**3.0** * **Provide details of the risks and discomforts associated with the research, in addition to standard care:**

The risks/discomforts of the study are minimal. The survey respondents will be asked to provide anonymous details regarding funding of clinical practice guidelines and organizational procedures relating to management of potential financial conflicts of interest.

**4.0** **Describe how you will manage and minimize risks and discomforts, as well as mitigate harm:**

All respondents will be anonymous, and we will attempt to obtain the same information from all producers of the 2012 guidelines within the National Guidelines Clearinghouse.

5.0 * **If your study has the potential to identify individuals who are upset, distressed, or disturbed, or warranting medical attention, describe the arrangements made to try to assist these individuals. If no arrangements have been made, please explain why:**
We do not foresee any participant becoming upset, distressed, or disturbed as a result of our survey.

## Reproductive Risk

*All questions preceded by a red asterisk (*) are required responses that map you to the application sections that are relevant to your study. All questions within the section to which you are mapped must be addressed.*

1.0 **Are there risks to any third parties associated with this study?**

**1.1 If Yes, please describe these risks and indicate what monitoring will be undertaken during the study and following the study conclusion:**

2.0 **Are there any reproductive risks associated with participation in the study?**

**2.1 If Yes, include a summary of the data regarding reproductive risks such as Teratogenicity or embryo toxicity of the study drug, risks related to breastfeeding or birth defects:**

3.0 **Are there risks to the fetus from men taking the study drug and fathering a child?**

**3.1 If Yes, fully describe these risks:**

4.0 **Are there risks to the female partner from men taking the study drug?**

**4.1 If Yes, fully describe these risks:**

5.0 **If there are risks to the fetus from men taking the study drug and fathering a child, will access to the records of the female partner and her baby be required?**

**5.1 If Yes, for what purpose?**

## Benefits Analysis

*All questions preceded by a red asterisk (*) are required responses that map you to the application sections that are relevant to your study. All questions within the section to which you are mapped must be addressed.*

1.0 * **Describe potential benefits of the proposed study to the participants. If there are no benefits, state this explicitly:**
There are no specific benefits to participants of the survey.

2.0 * **Describe the scientific, scholarly or societal benefits of the proposed research:**
Given the potential for far-reaching impact of guidelines on clinical practice, it is essential that these publications are free of significant COI. Above the level of individual authors, financial COI in the form of

funding for CPG producers may introduce significant bias; however, this issue is poorly established. A systematic analysis of potential COI related to funding at the guideline producer level is a key first step in defining the issue. Depending on the results of the analysis, there may be justification for an organized and uniform approach to minimizing and disclosing COI of this kind to allow end-users to reliably appraise clinical practice guidelines.

**3.0** **Benefits/Risks Analysis: Describe the relationship of benefits to risk of participation in the research:**

There is no significant benefit or risk to survey respondents.

## Participant Information

*All questions preceded by a red asterisk (*) are required responses that map you to the application sections that are relevant to your study. All questions within the section to which you are mapped must be addressed.*

**1.0** * **Describe the population that will be included in this study:**

The population is all clinical guidelines published in the National Guidelines Clearinghouse repository from Jan 1st, 2012- Dec. 31st, 2012. The population that will receive a survey are representatives for the organizations that publish the above guidelines.

**2.0** * **Describe and justify the inclusion criteria for participants:**

All clinical guidelines published in the National Guidelines Clearinghouse repository from Jan 1st, 2012- Dec. 31st, 2012. The inclusion criteria by date is a sample of convenience.

**3.0** **Describe and justify the exclusion criteria for participants, if applicable:**

Documents published as guidelines that do not contain any recommendations will be excluded.

**4.0** * **Will members of the research team be interacting with, or contacting participants, in person or electronically?**

(•) **Yes** ( ) No

Yes examples include: interventional studies, communication with participants, including web-based interaction, survey/questionnaire administration, qualitative studies, etc.

No examples include: secondary data analysis including health records review, database linkage, archival research, etc.

**5.0** Participants

* **5.1 How many participants do you expect to recruit (including controls, if applicable)?**

300

**5.2 Of these how many are controls, if applicable?**

N/A

**5.3 If this is a multi-site study, how many participants are expected to be enrolled by all investigators at all sites in the entire study? *(including controls, if applicable)***

**6.0** **Provide justification for sample size:**

We are using a sample of convenience. There are 374 guidelines published in 2012 in the National Guidelines Clearinghouse. There are multiple guidelines from a single producer, which may decrease the number of organizations surveyed to approximately 300.

7.0 * **Does the research specifically target aboriginal groups or communities?**

Yes **No** (selected)

## Recruit Potential Participants

1.0 **Recruitment**

**1.1 How will potential participants be identified? Describe how you will identify the people who will be approached for participation or screened for eligibility:**

The population is all clinical guidelines published in the National Guidelines Clearinghouse repository from Jan 1st, 2012- Dec. 31st, 2012. The population that will receive a survey are representatives for the organizations that publish the above guidelines.

**1.2 How will people obtain details about the research in order to make a decision about participating? *(select all that apply)***

Researchers will contact potential participants

**1.3 Provide the locations where the participants will be recruited (e.g. educational institutions, facilities in Alberta Health Services, etc.):**

By email survey.

2.0 **Pre-Existing Relationships**

**2.1 Will potential participants be recruited through pre-existing relationships with researchers?**

Yes **No** (selected)

**2.2 If Yes, identify the relationship between the researchers and participants that could compromise the freedom to decline. How will you ensure that there is no undue pressure on the potential participants to agree to participate?**

3.0 **Outline any other means by which participants will be recruited:**

N/A

4.0 **Will the study involve any of the following? *(select all that apply)***

None of the above

## Informed Consent Determination

*All questions preceded by a red asterisk (*) are required responses that map you to the application sections that are relevant to your study. All questions within the section to which you are mapped must be addressed.*

1.0 * **What type of consent will you be seeking? *(select all that apply)***

Consent from a competent adult

**1.1 Provide justification for requesting a Waiver of Consent *(minimal risk only)*:**

2.0 **How is consent to be indicated and documented? Select all that apply:**
Consent implied by specific action

**2.1 Except for "Signed consent form" use only, explain how the study information will be communicated and participant consent will be documented. Provide details for EACH of the option selected above:**
A consent statement will be included on the survey explicitly stating that by completing the survey the respondent is giving consent for the study.

3.0 Authorized Representative, Third Party Consent, Assent

**3.1 Explain why the participant is unable to give informed consent (e.g. young age, mental or physical condition, etc.):**
N/A

**3.2 Will the participant who is not competent to give full informed consent be asked to give assent?**
○ Yes ○ No

**Provide details. If applicable, attach a copy of the Assent form(s) in the Documentation section 'Assent Forms' at the end of the application:**
N/A

**3.3 In cases where participants (re)gain capacity to give informed consent during the study, how will they be asked to provide consent on their own behalf?**
N/A

4.0 **What assistance will be provided to participants, or those consenting on their behalf, who have special needs?**
N/A

5.0 * **When a participant wishes to end participation in the research or certain aspects of the research, describe what will occur:**
An email address will be provided with the survey to which they can write to request to withdraw from the study.

6.0 **Describe the circumstances and limitations of data withdrawal from the study, including the last point at which it can be done:**
The only limitation will be requests after proposed completion of data analysis in Oct. 2013.

7.0 **Will this study involve any group(s) where non-participants are present?**
○ Yes ● **No**

## Research Methods and Procedures

*All questions preceded by a red asterisk (*) are required responses that map you to the application sections that are relevant to your study. All questions within the section to which you are mapped must be addressed.*

**1.0** * **This study will involve the following: (*select all that apply*)**

- [ ] Use of deception or partial disclosure (not including double-blind procedures)
- [ ] Interviews (in-person, telephone, email, videoconferencing, chat rooms, etc.)
- [ ] Focus groups (including electronic/online focus groups)
- [x] **Surveys and questionnaires (including internet surveys and questionnaires)**
- [ ] Behavioral tasks
- [ ] Community-based research
- [ ] Participatory action research (participants are collaborators or co-researchers)
- [ ] Observational research (including internet-based observations)
- [ ] Sound or image (other than audio- or video-recordings of interviews; e.g., video recording of a dance performance; audio recording of pronunciations)
- [ ] Materials created by participants (e.g., artwork, writing samples, journals, software, etc.)
- [ ] Use of Psychology Research Participation System (RPS - requires separate registration with RPS)
- [ ] Food, nutrition, and nutraceuticals
- [ ] Drugs and natural health products
- [ ] Biologics and/or vaccines
- [ ] Medical devices
- [ ] Health and biological specimen collection (including use of previously collected specimens)
- [ ] Chart reviews
- [ ] Registries and databases (including biobanks)
- [ ] Radiation/diagnostic Imaging; any test or procedure that may involve exposure to radiation (including screening chest x-ray, MRI)
- [ ] Non-medical electro-mechanical devices
- [ ] Stem cell research (attach SCOC Approval in Documentation section "Other")
- [ ] None of the above

## Research Methods and Procedures (continued)

**1.0** * **Is this study a clinical trial?**

( ) Yes
(•) **No**

**2.0** **If you are using any tests in this study diagnostically, indicate the member(s) of the study team who will administer the measures/instruments:**

| Test Name | Test Administrator | Organization | Administrator's Qualifications |
|---|---|---|---|
| There are no items to display | | | |

**3.0** **If any test results could be interpreted diagnostically, how will these be reported back to the participants?**

**4.0** **What are the primary and secondary outcome variables that will be measured and how will the variables be measured?**

**4.1 Provide details about the data analysis method.*(1000 characters)***

**Archived Responses:**
**For clinical research only, describe any sub-studies associated with this application:**
N/A

## Interviews, Focus Groups, Surveys and Questionnaires

**1.0** **Are any of the questions potentially of a sensitive nature?**
◯Yes ◉**No**

**1.1 If Yes, provide details:**

**2.0** **If any data were released, could it reasonably place participants at risk of criminal or civil law suits?**
◯Yes ◉**No**

**2.1 If Yes, provide the justification for including such information in the study:**

**3.0** **Will you be capturing sound or images for the study?**
◯Yes ◉**No**

**3.1 If Yes, provide details:**

## Data Collection

*All questions preceded by a red asterisk (*) are required responses that map you to the application sections that are relevant to your study. All questions within the section to which you are mapped must be addressed.*

**1.0** * **Will the researcher or study team be able to identify any of the participants at any stage of the study?**
◉**Yes** ◯No

**2.0** **Will participants be recruited or their data be collected from Alberta Health Services or a data custodian as defined in the Alberta Health Information Act?**
◯Yes ◉**No**

**Important:** The Health Information Act requires a researcher to have a formal written agreement with the custodian of health information. This occurs after ethics approval.

**3.0** **Primary/raw data collected will be (check all that apply):**

**All personal identifying information removed** (anonymized)

**4.0** **If this study involves secondary use of data, list all original sources:**

Data on potential conflicts of interest and organizational procedures for management of potential conflict of interest will be obtained, where possible, from the published guideline or from the producer's website. Wherever this information is not available, a survey will be sent by email to the guideline producer.

**5.0** **In research where total anonymity and confidentiality cannot be guaranteed (e.g. where participants talk in a group) what measures will be taken to protect confidentiality to the degree possible, and what information will participants be given about limits to confidentiality?**

All email surveys will be anonymized and data will be stored on a single password-protected personal computer and all research data files will be encrypted and password-protected.

## Data Identifiers

*All questions preceded by a red asterisk (*) are required responses that map you to the application sections that are relevant to your study. All questions within the section to which you are mapped must be addressed.*

**1.0** * **Personal Identifiers: will you be collecting – at any time of the study, including recruitment of participants – any of the following *(check all that apply)*:**

Surname and first name

**If Other, describe:**

* **1.1 Provide a comprehensive rationale to explain why it is necessary to collect this information:**

The survey recipients' names will be collected in order to determine which recipients have responded and which will receive a reminder. Once the survey data is collected it will be anonymized.

**2.0** **Will you be collecting, assessing or disclosing at any time of the study, including recruitment of participants any of the following: *(check all that apply)***

There are no items to display

**If Other, describe:**

**2.1 Provide a comprehensive rationale to explain why it is necessary to collect this information:**

**3.0** **If identifying information will be removed at some point, when and how will this be done?**

**4.0** * **Specify what identifiable information will be RETAINED once data collection is complete, and explain why retention is necessary. Include the retention of master lists that link participant identifiers with de-identified data:**

A list of the participants' affiliated organizations with corresponding codes will be maintained to ensure that there is no duplication of data. Respondents' names or other personal information will not be retained.

**5.0** **If applicable, describe your plans to link the data in this study with data associated with other studies (e.g. within a data repository) or with data belonging to another organization:**

## Data Confidentiality and Privacy

*All questions preceded by a red asterisk (*) are required responses that map you to the application sections that are relevant to your study. All questions within the section to which you are mapped must be addressed.*

1.0 *** How will confidentiality of the data be maintained? Describe how the identity of participants will be protected both during and after the research:**

A list of survey recipients names and affiliated organizations will be maintained until the data collection phase is complete. From this point on a list of only the organizations will be maintained. Data will be stored on a single password-protected personal computer and all research data files will be encrypted and password-protected.

2.0 **How will the Principal Investigator ensure that all study personnel are aware of their responsibilities concerning participants' privacy and the confidentiality of their information?**

The principal investigator will have a meeting will all investigators to outline the responsibilities and measures to protect privacy and confidentiality of participants as outlined in this application.

3.0 **External Data Access**

3.1 *** Will identifiable data be transferred or made available to persons or agencies outside of the research team?**

○ Yes ● **No**

3.2 **If Yes, describe in detail what identifiable information will be released, to whom, why they need access, and under what condition. What safeguards, including encryption, will be used to protect the identity of participants and the privacy of their data?**

4.0 **Provide details if identifiable data will be leaving the institution, province or country, (e.g. member of research team in another institution or country, etc.):**

## Data Storage, Retention and Disposal

*All questions preceded by a red asterisk (*) are required responses that map you to the application sections that are relevant to your study. All questions within the section to which you are mapped must be addressed.*

1.0 *** Describe the electronic security provisions and the physical security provisions:**

Data will be stored on a single password-protected personal computer and all research data files will be encrypted and password-protected. No hardcopies of research materials with participant identifiers will be made.

2.0 *** Specify any plans for future use of the data. If this study involves the creation of research database or registry for future research use, please provide details:**

The data produced in this study will not be used for future research.

3.0 **If you plan to destroy your data, describe when and how that will be done and who will have access. Indicate your plans for the destruction of the identifiers at the earliest opportunity**

**consistent with the conduct of the research and/or clinical needs:**
Participant names will be deleted from the master list once the data collection period has completed.

## Documentation

Add documents in this section according to the headers, if applicable. Use "Other Documents" for any documents not specifically mentioned in the sections below. The document name *(and if applicable, version and date as reflected in the document)* you provide below will be listed on your approval letter.

**1.0 Recruitment Materials:**

**Attach supporting documents, naming them as you want them to appear in the approval letter:**

| Document Name | Document | Version | Document Date | Upload Date |
|---|---|---|---|---|
| There are no items to display | | | | |

**2.0 Letter of Initial Contact:**

**Attach supporting documents, naming them as you want them to appear in the approval letter:**

| | Document Name | Document | Version | Document Date | Upload Date |
|---|---|---|---|---|---|
| View | ethics intro letter.pdf | ethics intro letter.pdf | V.1 | 22 May 2013 | 22 May 2013 |

**3.0 Informed Consent / Information Document(s):**

**3.1 What is the reading level of the Informed Consent Form(s)?**
Consent is included in the intro letter attached above.

**3.2 Attach supporting documents, naming them as you want them to appear in the approval letter:**

| Document Name | Document | Version | Document Date | Upload Date |
|---|---|---|---|---|
| There are no items to display | | | | |

**4.0 Assent Forms:**

**Attach supporting documents, naming them as you want them to appear in the approval letter:**

| Document Name | Document | Version | Document Date | Upload Date |
|---|---|---|---|---|
| There are no items to display | | | | |

**5.0 Questionnaires, Cover Letters, Surveys, Tests, Interview Scripts, etc.**

**Attach supporting documents, naming them as you want them to appear in the approval letter:**

| | Document Name | Document | Version | Document Date | Upload Date |
|---|---|---|---|---|---|
| View | survey.pdf | survey.pdf | V.1 | 22 May 2013 | 22 May 2013 |

**6.0 Protocol:**

**Attach supporting documents, naming them as you want them to appear in the approval letter:**

| | Document Name | Document | Version | Document Date | Upload Date |
|---|---|---|---|---|---|
| View | protocol for ethics.pdf | protocol for ethics.pdf | V.1 | 22 May 2013 | 22 May 2013 |

**7.0 Investigator Brochures/Product Monographs (*Clinical Applications only*):**

**Attach supporting documents, naming them as you want them to appear in the approval letter:**

| Document Name | Document | Version | Document Date | Upload Date |
|---|---|---|---|---|
| There are no items to display | | | | |

**8.0 Health Canada No Objection Letter (NOL):**

**Attach supporting documents, naming them as you want them to appear in the approval letter:**

| Document Name | Document | Version | Document Date | Upload Date |
|---|---|---|---|---|
| There are no items to display | | | | |

**9.0 Confidentiality Agreement:**

**Attach supporting documents, naming them as you want them to appear in the approval letter:**

| Document Name | Document | Version | Document Date | Upload Date |
|---|---|---|---|---|
| There are no items to display | | | | |

**10.0 Conflict of Interest:**

**Attach supporting documents, naming them as you want them to appear in the approval letter:**

| Document Name | Document | Version | Document Date | Upload Date |
|---|---|---|---|---|
| There are no items to display | | | | |

**11.0 Other Documents:**

**Attach supporting documents, naming them as you want them to appear in the approval letter:**

| | Document Name | Document | Version | Document Date | Upload Date |
|---|---|---|---|---|---|
| View | BUDGET SUMMARY.pdf | BUDGET SUMMARY.pdf | V.1 | 22 May 2013 | 22 May 2013 |

**12.0 Electronic Re-submission Documentation:**

**Attach supporting documents:**

| Document Name | Document | Version | Document Date | Upload Date |
|---|---|---|---|---|
| There are no items to display | | | | |

**13.0 REB Service(s) Fee:**

**Attach supporting documents, naming them as you want them to appear in the approval letter:**

| Document Name | Document | Version | Document Date | Upload Date |
|---|---|---|---|---|
