# Supplementary material for: Financial Relationships between Organizations That Produce Clinical Practice Guidelines and the Biomedical Industry: A Cross-Sectional Study
Source: PLoS Med. 2016 May 31;13(5):e1002029. doi: 10.1371/journal.pmed.1002029 (PMC4887051; doi:10.1371/journal.pmed.1002029)
Supplement: S2 Text — (PDF) [file pmed.1002029.s003.pdf]

2014

Clinical Practice Guidelines: Policies for Managing Conflict of Interest

Dear Stakeholder,

We need your help.

We are conducting an international study to examine how organizations fund clinical guideline development and what procedures they use to minimize potential conflicts of interest. Your organization's experience with clinical guideline development is valuable and will help us and others understand how to manage this challenge.

Our survey is brief and can be answered in less than 5 minutes online at the following website:

https://www.surveymonkey.com/s/CPGConflictsofInterest

Your participation in this survey is voluntary, and your responses will be kept confidential. The survey has full ethics approval and your participation implies consent. If you are not the best person in your organization to answer this survey please email us the name of a more appropriate individual to contact.

If you have questions, do not hesitate to contact us using the following email: paul.campsall@albertahealthservices.ca

If you would like to decline participation, simply respond to this email with 'decline' in the subject line.

We appreciate your time and will be happy to share a summary of the results.

Thanks for your help!

Regards,

Dr. Sharon Straus

Department of Internal Medicine, University of Toronto

and Dr. Tom Stelfox

Dr. Paul Campsall

Conflicts of Interest - CPG

Department of Critical Care Medicine, University of Calgary

What is the name of the society/organization that you work with?

Please indicate the type of society/organization you work with

- ◯ Professional Association
- ◯ Governmental Organization
- ◯ Disease/Condition Interest Group
- ◯ Other

Other (please specify)

What is the approximate membership of your society/organization? (number of members)

Does your society/organization have a policy for managing potential conflicts of interest during guideline development?

- ◯ Yes
- ◯ No

If yes, is this policy accessible on your society/organization website?

- ◯ Yes
- ◯ No

Please provide the URL for where the policy can be located on the website.

Conflicts of Interest - CPG

Can you please provide us with a copy of the policy? Email policy to: Paul.Campsall@albertahealthservices.ca

Different policies for managing potential conflicts of interest during clinical guideline development have been proposed. Please indicate what best reflects the content of your organizational policy:

Committee members are required to disclose:

	Yes	No	Unsure	N/A
Financial conflicts of interest	○	○	○	○
Academic/intellectual conflicts of interest	○	○	○	○
Potential conflicts perceived as relevant to the guideline contents	○	○	○	○
All potential conflicts regardless of perceived relevance to the guideline contents	○	○	○	○

Composition of the guideline committee:

	Yes	No	Unsure	N/A
An attempt is made to recruit committee members without any conflicts	○	○	○	○
The majority of committee members must be free of conflicts	○	○	○	○
The committee chair must be free of potential conflicts	○	○	○	○
There is a standing committee within the society/organization (whose members are free of potential conflicts and includes at least one member that is not an employee or member of the board) that oversees institutional conflicts of interest	○	○	○	○

Managing conflicts during guideline development:

	Yes	No	Unsure	N/A
Committee members with conflicts are able to deliberate, draft or vote on related recommendations	○	○	○	○
Committee members are blinded to which companies financially contributed to guideline production (i.e. not aware of where the funds come from)	○	○	○	○
Potential committee member conflicts are reviewed prior to guideline production	○	○	○	○

Publishing guidelines and conflicts of interest:

	Yes	No	Unsure	N/A
Potential conflicts of interest of committee members are published in guidelines	○	○	○	○
The guidelines are peer reviewed by clinicians not involved in the clinical guideline production	○	○	○	○
Clinical guidelines are subject to independent review by the journal in which they are published	○	○	○	○

Conflicts of Interest - CPG

Role of industry partners in guideline development:

	Yes	No	Unsure	N/A
Industry partners are permitted to directly fund clinical guideline development	◯	◯	◯	◯
Industry partners may participate in the selection of clinical guideline committee members	◯	◯	◯	◯
Industry products (e.g., pharmaceuticals) are referred to in the clinical guidelines by their generic name	◯	◯	◯	◯
Industry partners are permitted to review clinical guidelines prior to release	◯	◯	◯	◯

Does your organization have a procedure for managing breakdowns in the conflict of interest policy during guideline development (i.e. policy not adhered to)?

◯ Yes

◯ No

◯ Unsure

◯ N/A

If yes, please provide a brief description of this procedure.

In your opinion, what elements of your organization's policy for managing conflicts of interest work well?

In your opinion, how could policies to manage conflicts of interest be improved?

Conflicts of Interest - CPG

What is the approximate total yearly revenue of your society/organization?

- <$1,000,000
- $1,000,000 - $5,000,000
- $5,000,000 - $10,000,000
- $10,000,000 - $25,000,000
- $25,000,000 - $50,000,000
- $50,000,000 - $100,000,000
- >$100,000,000
- Unsure

How is your organization funded? Select all that apply and indicate the approximate percentage if known.

	Approximate amount as percentage of operating budget
Governmental funds	
Income from organizational activities including membership dues, conference proceeds, journals including advertising, continuing education etc	
Charitable donations	
Donations from not for profit companies	
Donations from for-profit companies	

How does your organization fund guideline development? Select all that apply.

	Funding applied through general revenues	Funding directly applied to clinical guideline development
Governmental funds	☐	☐
Income from organizational activities (membership dues, conference proceeds, journals including advertising, continuing education etc.)	☐	☐
Charitable donations	☐	☐
Donations from not for profit companies	☐	☐
Donations from for-profit companies	☐	☐

Conclusion

Thank you very much for taking the time to complete the survey! We really appreciate your time and feedback! If you have any additional information please contact Paul Campsall at paul.campsall@albertahealthservices.ca

Thank you.
