# Supplementary material for: Financial Relationships between Organizations That Produce Clinical Practice Guidelines and the Biomedical Industry: A Cross-Sectional Study
Source: PLoS Med. 2016 May 31;13(5):e1002029. doi: 10.1371/journal.pmed.1002029 (PMC4887051; doi:10.1371/journal.pmed.1002029)
Supplement: S3 Text — (DOCX) [file pmed.1002029.s004.docx]

**S3 Text: Clinical Practice Guideline Bibliography**

1. ABM Clinical Protocol #25: Recommendations for preprocedural fasting for the breastfed infant: "NPO" Guidelines. *Breastfeed Med.* 2012;7(3):197-202.

2. KDOQI Clinical Practice Guideline for Diabetes and CKD: 2012 Update. *Am J Kidney Dis.* 2012;60(5):850-886.

3. ACOG Practice Bulletin Number 131: Screening for cervical cancer. *Obstet Gynecol.* 2012;120(5):1222-1238.

4. Practice bulletin no. 128: diagnosis of abnormal uterine bleeding in reproductive-aged women. *Obstet Gynecol.* 2012;120(1):197-206.

5. ACOG practice bulletin no. 127: Management of preterm labor. *Obstet Gynecol.* 2012;119(6):1308-1317.

6. Guiding principles for the care of older adults with multimorbidity: an approach for clinicians: American Geriatrics Society Expert Panel on the Care of Older Adults with Multimorbidity. *J Am Geriatr Soc.* 2012;60(10):E1-e25.

7. Prevention and control of influenza with vaccines: recommendations of the Advisory Committee on Immunization Practices (ACIP)--United States, 2012-13 influenza season. *MMWR Morb Mortal Wkly Rep.* 2012;61(32):613-618.

8. Update to CDC's Sexually transmitted diseases treatment guidelines, 2010: oral cephalosporins no longer a recommended treatment for gonococcal infections. *MMWR Morb Mortal Wkly Rep.* 2012;61(31):590-594.

9. Interim guidance for clinicians considering the use of preexposure prophylaxis for the prevention of HIV infection in heterosexually active adults. *MMWR Morb Mortal Wkly Rep.* 2012;61(31):586-589.

10. Updated CDC recommendations for the management of hepatitis B virus-infected health-care providers and students. *MMWR Recomm Rep.* 2012;61(Rr-3):1-12.

11. Update to CDC's U.S. Medical Eligibility Criteria for Contraceptive Use, 2010: revised recommendations for the use of hormonal contraception among women at high risk for HIV infection or infected with HIV. *MMWR Morb Mortal Wkly Rep.* 2012;61(24):449-452.

12. American Geriatrics Society updated Beers Criteria for potentially inappropriate medication use in older adults. *J Am Geriatr Soc.* 2012;60(4):616-631.

13. Practice advisory for perioperative visual loss associated with spine surgery: an updated report by the American Society of Anesthesiologists Task Force on Perioperative Visual Loss. *Anesthesiology.* 2012;116(2):274-285.

14. Practice guidelines for acute pain management in the perioperative setting: an updated report by the American Society of Anesthesiologists Task Force on Acute Pain Management. *Anesthesiology.* 2012;116(2):248-273.

15. Management of gynecologic issues in women with breast cancer. *American College of Obstetricians and Gynecologists.* 2012(ACOG practice bulletin; no. 126).

16. The 2012 hormone therapy position statement of: The North American Menopause Society. *Menopause.* 2012;19(3):257-271.

17. Management of perinatal mood disorders. A national clinical guideline. . 2012;SIGN publication; no. 127.

18. Management of vaginal discharge in non-genitourinary medicine settings. 2012.

19. Bacterial sepsis in pregnancy. 2012;Green-top guideline; no. 64a:14.

20. Bacterial sepsis following pregnancy. 2012;Green-top guideline; no. 64b:21.

21. The initial management of chronic pelvic pain. 2012;Green-top Guideline; no. 41:16.

22. The prevention of early-onset neonatal group B streptococcal disease. . 2012;Green-top guideline; no. 36:13.

23. Chronic fatigue syndrome/myalgic encephalomyelitis. A primer for clinical practitioners. . 2012.

24. Clinical Effectiveness Unit. Barrier methods for contraception and STI prevention. . 2012:28.

25. ACOG Practice Bulletin N. 129. Osteoporosis. *Obstet Gynecol.* 2012;120(3):718-734.

26. Preventive activities in older age. Guidelines for preventive activities in general practice, 8th edition. . 2012:28-33.

27. Osteoporosis. Guidelines for preventive activities in general practice, 8th edition. 2012:82-84.

28. Antithrombotics: indications and management. A national clinical guideline. . 2013:68.

29. Canadian Agency for Drugs and Technologies in Health (CADTH). Recommendations for antithrombotic agents for the prevention of stroke and systemic embolism in patients with atrial fibrillation. . 2013:16.

30. National Institute for Health and Clinical Excellence (NICE). Boceprevir for the treatment of genotype 1 chronic hepatitis C. 2012.

31. National Institute for Health and Clinical Excellence (NICE). Dasatinib, nilotinib and standard-dose imatinib for the first-line treatment of chronic myeloid leukaemia 2012.

32. National Institute for Health and Clinical Excellence (NICE). Eribulin for the treatment of locally advanced or metastatic breast cancer. 2012.

33. National Institute for Health and Clinical Excellence (NICE). Fingolimod for the treatment of highly active relapsing-remitting multiple sclerosis. 2012:55.

34. National Institute for Health and Clinical Excellence (NICE). Telaprevir for the treatment of genotype 1 chronic hepatitis C. 2012:47.

35. National Institute for Health and Clinical Excellence (NICE). Dabigatran etexilate for the prevention of stroke and systemic embolism in atrial fibrillation. 2012:44.

36. National Institute for Health and Clinical Excellence (NICE). Exenatide prolonged-release suspension for injection in combination with oral antidiabetic therapy for the treatment of type 2 diabetes. 2012:45.

37. National Institute for Health and Clinical Excellence (NICE). Pharmalgen for the treatment of bee and wasp venom allergy. 2012:34.

38. National Institute for Health and Clinical Excellence (NICE). Tocilizumab for the treatment of rheumatoid arthritis. 2012:55.

39. National Institute for Health and Clinical Excellence (NICE). Opioids in palliative care: safe and effective prescribing of strong opioids for pain in palliative care of adults. 2012;Clinical guideline; no. 140:23.

40. National Institute for Health and Clinical Excellence (NICE). Acute upper gastrointestinal bleeding: management. 2012;Clinical guideline; no. 141.

41. National Institute for Health and Clinical Excellence (NICE). Autism: recognition, referral, diagnosis and management of adults on the autism spectrum. . 2012;NICE clinical guideline; no. 142:57.

42. National Institute for Health and Clinical Excellence (NICE). Sickle cell acute painful episode: management of an acute painful sickle cell episode in hospital. 2012;NICE clinical guideline; no. 143:25.

43. National Institute for Health and Clinical Excellence (NICE). Venous thromboembolic diseases: the management of venous thromboembolic diseases and the role of thrombophilia testing. 2012;NICE clinical guideline; no. 144:40.

44. National Institute for Health and Clinical Excellence (NICE). Bevacizumab in combination with capecitabine for the first-line treatment of metastatic breast cancer. . 2012:48.

45. National Institute for Health and Clinical Excellence (NICE). Psoriasis: the assessment and management of psoriasis. . 2012:61.

46. National Institute for Health and Clinical Excellence (NICE). Denosumab for the prevention of skeletal-related events in adults with bone metastases from solid tumours. . 2012:56.

47. National Institute for Health and Clinical Excellence (NICE). Ivabradine for treating chronic heart failure. . 2012:49.

48. National Institute for Health and Clinical Excellence (NICE). Mannitol dry powder for inhalation for treating cystic fibrosis. . 2012:58.

49. National Institute for Health and Clinical Excellence (NICE). Vemurafenib for treating locally advanced or metastatic BRAF V600 mutation-positive malignant melanoma. . 2012:45.

50. National Institute for Health and Clinical Excellence (NICE). Ipilimumab for previously treated advanced (unresectable or metastatic) melanoma. . 2012:45.

51. National Institute for Health and Clinical Excellence (NICE). Crohn's disease: management in adults, children and young people. . 2012:34.

52. National Institute for Health and Clinical Excellence (NICE). Alteplase for treating acute ischaemic stroke (review of technology appraisal guidance 122). . 2013:37.

53. Scottish Intercollegiate Guidelines Network (SIGN). Antithrombotics: indications and management. A national clinical guideline. . 2013:68.

54. Allen VM, Yudin MH, Bouchard C, et al. Management of group B streptococcal bacteriuria in pregnancy. *J Obstet Gynaecol Can.* 2012;34(5):482-486.

55. Alonso-Coello P, Bellmunt S, McGorrian C, et al. Antithrombotic therapy in peripheral artery disease: Antithrombotic Therapy and Prevention of Thrombosis, 9th ed: American College of Chest Physicians Evidence-Based Clinical Practice Guidelines. *Chest.* 2012;141(2 Suppl):e669S-690S.

56. American Academy of Family Physicians. Summary of Recommendations for Clinical Preventive Services. 2012.

57. Andersen PM, Abrahams S, Borasio GD, et al. EFNS guidelines on the clinical management of amyotrophic lateral sclerosis (MALS)--revised report of an EFNS task force. *Eur J Neurol.* 2012;19(3):360-375.

58. Apfelbaum JL, Connis RT, Nickinovich DG, et al. Practice advisory for preanesthesia evaluation: an updated report by the American Society of Anesthesiologists Task Force on Preanesthesia Evaluation. *Anesthesiology.* 2012;116(3):522-538.

59. Armstrong MJ, Miyasaki JM. Evidence-based guideline: pharmacologic treatment of chorea in Huntington disease: report of the guideline development subcommittee of the American Academy of Neurology. *Neurology.* 2012;79(6):597-603.

60. Arsenault D, Brenn M, Kim S, et al. A.S.P.E.N. Clinical Guidelines: hyperglycemia and hypoglycemia in the neonate receiving parenteral nutrition. *JPEN J Parenter Enteral Nutr.* 2012;36(1):81-95.

61. Aurora RN, Kristo DA, Bista SR, et al. The treatment of restless legs syndrome and periodic limb movement disorder in adults--an update for 2012: practice parameters with an evidence-based systematic review and meta-analyses: an American Academy of Sleep Medicine Clinical Practice Guideline. *Sleep.* 2012;35(8):1039-1062.

62. Aurora RN, Lamm CI, Zak RS, et al. Practice parameters for the non-respiratory indications for polysomnography and multiple sleep latency testing for children. *Sleep.* 2012;35(11):1467-1473.

63. Balasegaram S, Potter AL, Grynszpan D, et al. Guidelines for the public health management of typhoid and paratyphoid in England: practice guidelines from the National Typhoid and Paratyphoid Reference Group. *J Infect.* 2012;65(3):197-213.

64. Bates SM, Greer IA, Middeldorp S, Veenstra DL, Prabulos AM, Vandvik PO. VTE, thrombophilia, antithrombotic therapy, and pregnancy: Antithrombotic Therapy and Prevention of Thrombosis, 9th ed: American College of Chest Physicians Evidence-Based Clinical Practice Guidelines. *Chest.* 2012;141(2 Suppl):e691S-736S.

65. Bates SM, Jaeschke R, Stevens SM, et al. Diagnosis of DVT: Antithrombotic Therapy and Prevention of Thrombosis, 9th ed: American College of Chest Physicians Evidence-Based Clinical Practice Guidelines. *Chest.* 2012;141(2 Suppl):e351S-418S.

66. Baughman RP, Meyer KC, Nathanson I, et al. Monitoring of nonsteroidal immunosuppressive drugs in patients with lung disease and lung transplant recipients: American College of Chest Physicians evidence-based clinical practice guidelines. *Chest.* 2012;142(5):e1S-e111S.

67. Becker GJ, Wheeler DC, Zeeuw DD. KDIGO clinical practice guideline for the management of blood pressure in chronic kidney disease. *Kidney Int.* 2012;2:S337-414.

68. Bennett DL NJ, Weissman BN, Kransdorf MJ, Appel M, Bencardino JT, Fries IB, Hayes CW, Hochman MG, Jacobson JA, Luchs JS, Math KR, Murphey MD, Newman JS, Rubin DA, Scharf SC, Small KM, Expert Panel on Musculoskeletal Imaging. . ACR Appropriateness Criteria® nontraumatic knee pain. . 2012:10.

69. Bentley J. Colposcopic management of abnormal cervical cytology and histology. *J Obstet Gynaecol Can.* 2012;34(12):1188-1206.

70. Berglund L, Brunzell JD, Goldberg AC, et al. Evaluation and treatment of hypertriglyceridemia: an Endocrine Society clinical practice guideline. *J Clin Endocrinol Metab.* 2012;97(9):2969-2989.

71. Birbeck GL, French JA, Perucca E, et al. Evidence-based guideline: Antiepileptic drug selection for people with HIV/AIDS: report of the Quality Standards Subcommittee of the American Academy of Neurology and the Ad Hoc Task Force of the Commission on Therapeutic Strategies of the International League Against Epilepsy. *Neurology.* 2012;78(2):139-145.

72. Broderick DF WFI, Cornelius RS, Aiken AH, Angtuaco EJ, Brown DC, Holloway K, McConnell CT Jr, Mechtler LL, Rosenow JM, Roth CJ, Slavin K, Expert Panel on Neurologic Imaging. . ACR Appropriateness Criteria® ataxia. 2012.

73. Brophy GM, Bell R, Claassen J, et al. Guidelines for the evaluation and management of status epilepticus. *Neurocrit Care.* 2012;17(1):3-23.

74. Brown K GA, Mohammed TL, Kirsch J, Chung JH, Dyer DS, Ginsburg ME, Heitkamp DE, Kanne JP, Kazerooni EA, Ketai LH, Parker JA, Ravenel JG, Saleh AG, Shah RD, Steiner RM, Suh RD, Expert Panel on Thoracic Imaging. . ACR Appropriateness Criteria® pulmonary hypertension. . 2012:10.

75. Burke CT RCJ, Lorenz JM, Darcy MD, Fidelman N, Hohenwalter EJ, Kinney TB, Kolbeck KJ, Kouri BE, Nair AV, Rochon PJ, Shaw H, Vatakencherry G, Expert Panel on Interventional Radiology. . ACR Appropriateness Criteria® radiologic management of uterine leiomyomas. . 2012:8.

76. Calkins H, Kuck KH, Cappato R, et al. 2012 HRS/EHRA/ECAS expert consensus statement on catheter and surgical ablation of atrial fibrillation: recommendations for patient selection, procedural techniques, patient management and follow-up, definitions, endpoints, and research trial design: a report of the Heart Rhythm Society (HRS) Task Force on Catheter and Surgical Ablation of Atrial Fibrillation. Developed in partnership with the European Heart Rhythm Association (EHRA), a registered branch of the European Society of Cardiology (ESC) and the European Cardiac Arrhythmia Society (ECAS); and in collaboration with the American College of Cardiology (ACC), American Heart Association (AHA), the Asia Pacific Heart Rhythm Society (APHRS), and the Society of Thoracic Surgeons (STS). Endorsed by the governing bodies of the American College of Cardiology Foundation, the American Heart Association, the European Cardiac Arrhythmia Society, the European Heart Rhythm Association, the Society of Thoracic Surgeons, the Asia Pacific Heart Rhythm Society, and the Heart Rhythm Society. *Heart Rhythm.* 2012;9(4):632-696.e621.

77. Cancer. NCCf. Neutropenic sepsis: prevention and management of neutropenic sepsis in cancer patients. . 2012:31.

78. Cantrill SV, Brown MD, Carlisle RJ, et al. Clinical policy: critical issues in the prescribing of opioids for adult patients in the emergency department. *Ann Emerg Med.* 2012;60(4):499-525.

79. Cartwright MS, Hobson-Webb LD, Boon AJ, et al. Evidence-based guideline: neuromuscular ultrasound for the diagnosis of carpal tunnel syndrome. *Muscle Nerve.* 2012;46(2):287-293.

80. Carty SE, Doherty GM, Inabnet WB, 3rd, et al. American Thyroid Association statement on the essential elements of interdisciplinary communication of perioperative information for patients undergoing thyroid cancer surgery. *Thyroid.* 2012;22(4):395-399.

81. National Clinical Guideline Centre. Lower limb peripheral arterial disease: diagnosis and management. . 2012:28.

82. National Clinical Guideline Centre. Osteoporosis: assessing the risk of fragility fracture. . 2012:22.

83. National Clinical Guideline Centre. Urinary incontinence in neurological disease. Management of lower urinary tract dysfunction in neurological disease. . 2012:40.

84. National Clinical Guideline Centre. Headaches: diagnosis and management of headaches in young people and adults. . 2012:38.

85. Chalasani N, Younossi Z, Lavine JE, et al. The diagnosis and management of non-alcoholic fatty liver disease: practice Guideline by the American Association for the Study of Liver Diseases, American College of Gastroenterology, and the American Gastroenterological Association. *Hepatology.* 2012;55(6):2005-2023.

86. Chang GJ, Kaiser AM, Mills S, Rafferty JF, Buie WD. Practice parameters for the management of colon cancer. *Dis Colon Rectum.* 2012;55(8):831-843.

87. Chow AW, Benninger MS, Brook I, et al. IDSA clinical practice guideline for acute bacterial rhinosinusitis in children and adults. *Clin Infect Dis.* 2012;54(8):e72-e112.

88. Cohen MI, Triedman JK, Cannon BC, et al. PACES/HRS expert consensus statement on the management of the asymptomatic young patient with a Wolff-Parkinson-White (WPW, ventricular preexcitation) electrocardiographic pattern: developed in partnership between the Pediatric and Congenital Electrophysiology Society (PACES) and the Heart Rhythm Society (HRS). Endorsed by the governing bodies of PACES, HRS, the American College of Cardiology Foundation (ACCF), the American Heart Association (AHA), the American Academy of Pediatrics (AAP), and the Canadian Heart Rhythm Society (CHRS). *Heart Rhythm.* 2012;9(6):1006-1024.

89. Cornelius RS MJ, Wippold FJ II, Aiken AH, Angtuaco EJ, Berger KL, Brown DC, Davis PC, McConnell CT Jr, Mechtler LL, Nussenbaum B, Roth CJ, Seidenwurm DJ, Expert Panel on Neurologic Imaging. . ACR Appropriateness Criteria® sinonasal disease. . 2012:7.

90. Cupler EJ, Berger KI, Leshner RT, et al. Consensus treatment recommendations for late-onset Pompe disease. *Muscle Nerve.* 2012;45(3):319-333.

91. Daffner RH WB, Wippold FJ II, Angtuaco EJ, Appel M, Berger KL, Cornelius RS, Douglas AC, Fries IB, Hayes CW, Holly L, Mechtler LL, Prall JA, Rubin DA, Ward RJ, Waxman AD, Expert Panels on Musculoskeletal and Neurologic Imaging. ACR Appropriateness Criteria® suspected spine trauma. 2012:20.

92. Darragh TM, Colgan TJ, Cox JT, et al. The Lower Anogenital Squamous Terminology Standardization Project for HPV-Associated Lesions: background and consensus recommendations from the College of American Pathologists and the American Society for Colposcopy and Cervical Pathology. *Arch Pathol Lab Med.* 2012;136(10):1266-1297.

93. Daubert JC, Saxon L, Adamson PB, et al. 2012 EHRA/HRS expert consensus statement on cardiac resynchronization therapy in heart failure: implant and follow-up recommendations and management. *Heart Rhythm.* 2012;9(9):1524-1576.

94. Davis PC WFI, Cornelius RS, Aiken AH, Angtuaco EJ, Berger KL, Broderick DF, Brown DC, Douglas AC, McConnell CT Jr, Mechtler LL, Prall JA, Raksin PB, Roth CJ, Seidenwurm DJ, Smirniotopoulos JG, Waxman AD, Coley BD, Expert Panel on Neurologic Imaging. ACR Appropriateness Criteria® head trauma. 2012:14.

95. Davis R, Jones JS, Barocas DA, et al. Diagnosis, evaluation and follow-up of asymptomatic microhematuria (AMH) in adults: AUA guideline. *The Journal of urology.* 2012;188(6):2473-2481.

96. Department of Health and Human Services (U.S.). Guidelines for the use of antiretroviral agents in HIV-1-infected adults and adolescents. 2012.

97. Desjardins B, Dill KE, Flamm SD, et al. ACR Appropriateness Criteria® pulsatile abdominal mass, suspected abdominal aortic aneurysm. *The international journal of cardiovascular imaging.* 2013;29(1):177-183.

98. Dighe M RE, Casalino D, Bishoff JT, Blaufox MD, Coursey CA, Eberhardt SC, Goldfarb S, Harvin HJ, Lazarus E, Leyendecker JR, Lockhart ME, Nikolaidis P, Oto A, Porter C, Sheth S, Vikram R, Expert Panel on Urologic Imaging. . ACR Appropriateness Criteria® renal transplant dysfunction. . 2012:7.

99. Dignan FL, Amrolia P, Clark A, et al. Diagnosis and management of chronic graft-versus-host disease. *Br J Haematol.* 2012;158(1):46-61.

100. Dignan FL, Clark A, Amrolia P, et al. Diagnosis and management of acute graft-versus-host disease. *Br J Haematol.* 2012;158(1):30-45.

101. Dignan FL, Scarisbrick JJ, Cornish J, et al. Organ-specific management and supportive care in chronic graft-versus-host disease. *Br J Haematol.* 2012;158(1):62-78.

102. Dill KE RF, Desjardins B, Flamm SD, Francois CJ, Gerhard-Herman MD, Kalva SP, Mansour MA, Mohler ER III, Oliva IB, Schenker MP, Weiss C, Expert Panel on Vascular Imaging. . ACR Appropriateness Criteria® claudication -- suspected vascular etiology. . 2012:7.

103. Dillman JR CB, Karmazyn B, Binkovitz LA, Dempsey ME, Dory CE, Garber M, Hayes LL, Meyer JS, Milla SS, Paidas C, Raske ME, Rigsby CK, Strouse PJ, Wootton-Gorges SL, Expert Panel on Pediatric Imaging. . ACR Appropriateness Criteria® hematuria -- child. . 2012:9.

104. Dohle GR AS, Bettocchi C, Kliesch S, Punab M, de Ronde W. . Guidelines on male hypogonadism.

. 2012:28.

105. Dory CE CB, Karmazyn B, Charron M, Dempsey ME, Dillman JR, Garber M, Hayes LL, Holloway K, Milla SS, Raske ME, Rice HE, Rigsby CK, Rosenow JM, Strouse PJ, Westra SJ, Wootton-Gorges SL, Expert Panel on Pediatric Imaging. ACR Appropriateness Criteria® seizures -- child. . 2012:9.

106. Douketis JD, Spyropoulos AC, Spencer FA, et al. Perioperative management of antithrombotic therapy: Antithrombotic Therapy and Prevention of Thrombosis, 9th ed: American College of Chest Physicians Evidence-Based Clinical Practice Guidelines. *Chest.* 2012;141(2 Suppl):e326S-350S.

107. Draycott T, Fox R, Montague I. Royal College of Obstetricians and Gynaecologists (RCOG). Shoulder Dystocia. Clinical Guideline No. 42. London: RCOG Press; 2005.

108. Dunn S, Guilbert E. Emergency contraception. *J Obstet Gynaecol Can.* 2012;34(9):870-878.

109. Dyer DS MT, Kirsch J, Amorosa JK, Brown K, Chung JH, Ginsburg ME, Heitkamp DE, Kanne JP, Kazerooni EA, Ketai LH, Parker JA, Ravenel JG, Saleh AG, Shah RD, Expert Panel on Thoracic Imaging. . ACR Appropriateness Criteria® chronic dyspnea - suspected pulmonary origin. . 2012:5.

110. Eberhardt SC CS, Casalino DD, Merrick G, Frank SJ, Gottschalk AR, Leyendecker JR, Nguyen PL, Oto A, Porter C, Remer EM, Rosenthal SA, Expert Panels on Urologic Imaging and Radiation Oncology-Prostate. . ACR Appropriateness Criteria® prostate cancer -- pretreatment detection, staging, and surveillance. 2012:12.

111. Engeler D BA, Elneil S, Hughes J, Messelink EJ, Oliveira P, van Ophoven A, de C. Williams AC. . Urological aspects of chronic pelvic pain: Guidelines on chronic pelvic pain. . 2012:28-78.

112. Engeler D BA, Elneil S, Hughes J, Messelink EJ, Oliveira P, van Ophoven A, de C. Williams AC. . Gynaecological aspects of chronic pelvic pain. Guidelines on chronic pelvic pain. . 2012:79-85.

113. Engeler D BA, Elneil S, Hughes J, Messelink EJ, Oliveira P, van Ophoven A, de C. Williams AC. . Gastrointestinal aspects of chronic pelvic pain. Guidelines on chronic pelvic pain. . 2012: 85-90.

114. Engeler D BA, Elneil S, Hughes J, Messelink EJ, Oliveira P, van Ophoven A, de C. Williams AC. . Peripheral nerve pain syndromes. Guidelines on chronic pelvic pain. . 2012:90-100.

115. Engeler D BA, Elneil S, Hughes J, Messelink EJ, Oliveira P, van Ophoven A, de C. Williams AC. . Sexological aspects of chronic pelvic pain. Guidelines on chronic pelvic pain. . 2012:100-108.

116. Engeler D BA, Elneil S, Hughes J, Messelink EJ, Oliveira P, van Ophoven A, de C. Williams AC. . Psychological aspects of chronic pelvic pain. Guidelines on chronic pelvic pain. . 2012:109-116.

117. Engeler D BA, Elneil S, Hughes J, Messelink EJ, Oliveira P, van Ophoven A, de C. Williams AC. . Pelvic floor function and chronic pelvic pain. Guidelines on chronic pelvic pain. . 2012:116-122.

118. Engeler D BA, Elneil S, Hughes J, Messelink EJ, Oliveira P, van Ophoven A, de C. Williams AC. General treatment of chronic pelvic pain. Guidelines on chronic pelvic pain. . 2012:122-130.

119. Evans JA, Early DS, Fukami N, et al. The role of endoscopy in Barrett's esophagus and other premalignant conditions of the esophagus. *Gastrointest Endosc.* 2012;76(6):1087-1094.

120. National Institute for Health and Care Excellence. Infection: Prevention and Control of Healthcare-Associated Infections in Primary and Community Care: Partial Update of NICE Clinical Guideline 2. 2012(Appendix N). Accessed Mar.

121. Falck-Ytter Y, Francis CW, Johanson NA, et al. Prevention of VTE in orthopedic surgery patients: Antithrombotic Therapy and Prevention of Thrombosis, 9th ed: American College of Chest Physicians Evidence-Based Clinical Practice Guidelines. *Chest.* 2012;141(2 Suppl):e278S-325S.

122. Fallon EM, Nehra D, Potemkin AK, et al. A.S.P.E.N. clinical guidelines: nutrition support of neonatal patients at risk for necrotizing enterocolitis. *JPEN J Parenter Enteral Nutr.* 2012;36(5):506-523.

123. Ferraris VA, Saha SP, Oestreich JH, et al. 2012 update to the Society of Thoracic Surgeons guideline on use of antiplatelet drugs in patients having cardiac and noncardiac operations. *Ann Thorac Surg.* 2012;94(5):1761-1781.

124. Fihn SD, Gardin JM, Abrams J, et al. 2012 ACCF/AHA/ACP/AATS/PCNA/SCAI/STS Guideline for the diagnosis and management of patients with stable ischemic heart disease: a report of the American College of Cardiology Foundation/American Heart Association Task Force on Practice Guidelines, and the American College of Physicians, American Association for Thoracic Surgery, Preventive Cardiovascular Nurses Association, Society for Cardiovascular Angiography and Interventions, and Society of Thoracic Surgeons. *J Am Coll Cardiol.* 2012;60(24):e44-e164.

125. Filippi M, Agosta F, Barkhof F, et al. EFNS task force: the use of neuroimaging in the diagnosis of dementia. *Eur J Neurol.* 2012;19(12):e131-140, 1487-1501.

126. Francois CJ KJ, Rybicki FJ, Ray CE Jr, Bandyk DF, Burke CT, Dill KE, Gerhard-Herman MD, Hanley M, Hohenwalter EJ, Mohler ER III, Rochon PJ, Schenker MP, Expert Panel on Vascular Imaging and Interventional Radiology. ACR Appropriateness Criteria® abdominal aortic aneurysm: interventional planning and follow-up. 2012:8.

127. Gaffney DK EB, Jhingran A, Mayr NA, Puthawala AA, Cardenes HR, Elshaikh MA, Gullet N, Kidd E, Lee LJ, Moore D, Rao GG, Small W Jr, Varia MA, Wahl AO, Wolfson AH, Yashar CM, Yuh W, Expert Panel on Radiation Oncology-Gynecology. . ACR Appropriateness Criteria® advanced cervical cancer. 2012:8.

128. Garber JR, Cobin RH, Gharib H, et al. Clinical practice guidelines for hypothyroidism in adults: cosponsored by the American Association of Clinical Endocrinologists and the American Thyroid Association. *Endocr Pract.* 2012;18(6):988-1028.

129. Gardner C, Wylie-Rosett J, Gidding SS, et al. Nonnutritive sweeteners: current use and health perspectives: a scientific statement from the American Heart Association and the American Diabetes Association. *Circulation.* 2012;126(4):509-519.

130. Geoffrion R. Treatments for overactive bladder: focus on pharmacotherapy. *J Obstet Gynaecol Can.* 2012;34(11):1092-1101.

131. Gillis AM, Russo AM, Ellenbogen KA, et al. HRS/ACCF expert consensus statement on pacemaker device and mode selection. Developed in partnership between the Heart Rhythm Society (HRS) and the American College of Cardiology Foundation (ACCF) and in collaboration with the Society of Thoracic Surgeons. *Heart Rhythm.* 2012;9(8):1344-1365.

132. Go CY, Mackay MT, Weiss SK, et al. Evidence-based guideline update: medical treatment of infantile spasms. Report of the Guideline Development Subcommittee of the American Academy of Neurology and the Practice Committee of the Child Neurology Society. *Neurology.* 2012;78(24):1974-1980.

133. Gormley EA, Lightner DJ, Burgio KL, et al. Diagnosis and treatment of overactive bladder (non-neurogenic) in adults: AUA/SUFU guideline. *The Journal of urology.* 2012;188(6 Suppl):2455-2463.

134. Gould MK, Garcia DA, Wren SM, et al. Prevention of VTE in nonorthopedic surgical patients: Antithrombotic Therapy and Prevention of Thrombosis, 9th ed: American College of Chest Physicians Evidence-Based Clinical Practice Guidelines. *Chest.* 2012;141(2 Suppl):e227S-277S.

135. Griggs JJ, Mangu PB, Anderson H, et al. Appropriate chemotherapy dosing for obese adult patients with cancer: American Society of Clinical Oncology clinical practice guideline. *Journal of Clinical Oncology.* 2012;30(13):1553-1561.

136. Gronseth GS, Paduga R. Evidence-based guideline update: steroids and antivirals for Bell palsy: report of the Guideline Development Subcommittee of the American Academy of Neurology. *Neurology.* 2012;79(22):2209-2213.

137. Clinical Effectiveness Group. UK national guidelines on the management of adult and adolescent complainants of sexual assault 2011. 2012.

138. Group. KDIGOKGW. KDIGO clinical practice guideline for glomerulonephritis. . *Kidney Int Suppl. .* 2012;2(2):139-274.

139. Hahn BH, McMahon MA, Wilkinson A, et al. American College of Rheumatology guidelines for screening, treatment, and management of lupus nephritis. *Arthritis Care Res (Hoboken).* 2012;64(6):797-808.

140. Hahn SA, Lavonas EJ, Mace SE, Napoli AM, Fesmire FM. Clinical policy: Critical issues in the initial evaluation and management of patients presenting to the emergency department in early pregnancy. *Ann Emerg Med.* 2012;60(3):381-390.e328.

141. Harris RD JM, Glanc P, Brown DL, Dubinsky T, Harisinghani MG, Khati NJ, Kim YB, Mitchell DG, Pandharipande PV, Pannu HK, Podrasky AE, Royal HD, Shipp TD, Siegel CL, Simpson L, Wall DJ, Wong-You-Cheong JJ, Zelop CM, Expert Panel on Women's Imaging. . ACR Appropriateness Criteria® clinically suspected adnexal mass. 2012:13.

142. Harvey JA MM, Newell MS, Bailey L, Barke LD, D'Orsi C, Hayes MK, Jokich PM, Lee S, Lehman CD, Mainiero MB, Mankoff DA, Patel SB, Reynolds HE, Sutherland ML, Haffty BG, Expert Panel on Breast Imaging. ACR Appropriateness Criteria® palpable breast masses. 2012:16.

143. Harvin HJ CD, Remer EM, Bishoff JT, Coursey CA, Dighe M, Eberhardt SC, Goldfarb S, Lazarus E, Leyendecker JR, Lockhart ME, Majd M, Nikolaidis P, Oto A, Porter C, Ramchandani P, Sheth S, Vikram R, Expert Panel on Urologic Imaging. . ACR Appropriateness Criteria® renovascular hypertension. . 2012:10.

144. Hayes LL CB, Karmazyn B, Dempsey-Robertson ME, Dillman JR, Dory CE, Garber M, Keller MS, Kulkarni AV, Meyer JS, Milla SS, Myseros JS, Paidas C, Raske ME, Rigsby CK, Strouse PJ, Wootton-Gorges SL, Expert Panel on Pediatric Imaging. . ACR Appropriateness Criteria® headache - child. 2012:8.

145. National Collaborating Centre for Women's and Children's Health. Antibiotics for early-onset neonatal infection. 2012.

146. National Collaborating Centre for Women's and Children's Health. Spasticity in children and young people with non-progressive brain disorders. Management of spasticity and co-existing motor disorders and their early musculoskeletal complications. . 2012:54

147. National Collaborating Centre for Women's and Children's Health. Ectopic pregnancy and miscarriage. Diagnosis and initial management in early pregnancy of ectopic pregnancy and miscarriage. . 2012:36.

148. Hochberg MC, Altman RD, April KT, et al. American College of Rheumatology 2012 recommendations for the use of nonpharmacologic and pharmacologic therapies in osteoarthritis of the hand, hip, and knee. *Arthritis Care Res (Hoboken).* 2012;64(4):465-474.

149. Holbrook A, Schulman S, Witt DM, et al. Evidence-based management of anticoagulant therapy: Antithrombotic Therapy and Prevention of Thrombosis, 9th ed: American College of Chest Physicians Evidence-Based Clinical Practice Guidelines. *Chest.* 2012;141(2 Suppl):e152S-184S.

150. Holland S, Silberstein SD, Freitag F, Dodick DW, Argoff C, Ashman E. Evidence-based guideline update: NSAIDs and other complementary treatments for episodic migraine prevention in adults: report of the Quality Standards Subcommittee of the American Academy of Neurology and the American Headache Society. *Neurology.* 2012;78(17):1346-1353.

151. Horst KC HB, Harris EE, Bailey L, Bellon JR, Carey L, Freedman GM, Goyal S, Halyard MY, MacDonald SM, Moran MS, Suh WW, Expert Panel on Radiation Oncology - Breast. ACR Appropriateness Criteria® postmastectomy radiotherapy. 2012:15.

152. Hosseinzadeh K RE, Leyendecker JR, Eberhardt SC, Friedman B, Fulgham PF, Goldfarb S, Hartman MS, Lazarus E, Lockhart ME, Majd M, Oto A, Porter C, Sudakoff GS, Verma S, Expert Panel on Urologic Imaging. . ACR Appropriateness Criteria® hematospermia. . 2012:6.

153. Hull J, Aniapravan R, Chan E, et al. British Thoracic Society guideline for respiratory management of children with neuromuscular weakness. *Thorax.* 2012;67 Suppl 1:i1-40.

154. Hwang JH, Fisher DA, Ben-Menachem T, et al. The role of endoscopy in the management of acute non-variceal upper GI bleeding. *Gastrointest Endosc.* 2012;75(6):1132-1138.

155. Jacobi J, Bircher N, Krinsley J, et al. Guidelines for the use of an insulin infusion for the management of hyperglycemia in critically ill patients. *Crit Care Med.* 2012;40(12):3251-3276.

156. Kahn SR, Lim W, Dunn AS, et al. Prevention of VTE in nonsurgical patients: Antithrombotic Therapy and Prevention of Thrombosis, 9th ed: American College of Chest Physicians Evidence-Based Clinical Practice Guidelines. *Chest.* 2012;141(2 Suppl):e195S-226S.

157. Kanne JP, Jensen LE, Mohammed T-LH, et al. ACR Appropriateness Criteria® radiographically detected solitary pulmonary nodule. *Journal of thoracic imaging.* 2013;28(1):W1-W3.

158. Karmazyn B CB, Dempsey-Robertson ME, Dillman JR, Dory CE, Garber M, Hadley JA, Hayes LL, Keller MS, Meyer JS, Milla SS, Paidas C, Raske ME, Rigsby CK, Strouse PJ, Wootton-Gorges SL, Expert Panel on Pediatric Imaging. . ACR Appropriateness Criteria® sinusitis - child. 2012:7.

159. Karmazyn B CB, Binkovitz LA, Dempsey-Robertson ME, Dillman JR, Dory CE, Garber M, Hayes LL, Keller MS, Meyer JS, Milla SS, Paidas C, Raske ME, Rigsby CK, Strouse PJ, Wootton-Gorges SL, Expert Panel on Pediatric Imaging. . ACR Appropriateness Criteria® urinary tract infection - child. . 2012:8.

160. Kearon C, Akl EA, Comerota AJ, et al. Antithrombotic therapy for VTE disease: Antithrombotic Therapy and Prevention of Thrombosis, 9th ed: American College of Chest Physicians Evidence-Based Clinical Practice Guidelines. *Chest.* 2012;141(2 Suppl):e419S-494S.

161. Keeling D, Mackie I, Moore GW, Greer IA, Greaves M. Guidelines on the investigation and management of antiphospholipid syndrome. *Br J Haematol.* 2012;157(1):47-58.

162. Khanna D, Fitzgerald JD, Khanna PP, et al. 2012 American College of Rheumatology guidelines for management of gout. Part 1: systematic nonpharmacologic and pharmacologic therapeutic approaches to hyperuricemia. *Arthritis Care Res (Hoboken).* 2012;64(10):1431-1446.

163. Khanna D, Khanna PP, Fitzgerald JD, et al. 2012 American College of Rheumatology guidelines for management of gout. Part 2: therapy and antiinflammatory prophylaxis of acute gouty arthritis. *Arthritis Care Res (Hoboken).* 2012;64(10):1447-1461.

164. Khwaja A. KDIGO clinical practice guidelines for acute kidney injury. *The Nephron journals.* 2012;120(4).

165. Kidd E MD, Varia MA, Gaffney DK, Cardenes HR, Elshaikh MA, Erickson B, Jhingran A, Lee LJ, Mayr NA, Puthawala AA, Rao GG, Small W Jr, Wahl AO, Wolfson AH, Yashar CM, Yuh W, Expert Panel on Radiation Oncology-Gynecology. . ACR Appropriateness Criteria® management of locoregionally advanced squamous cell carcinoma of the vulva. . 2012:12.

166. Kinney TB AH, Ray CE Jr, Lorenz JM, Burke CT, Darcy MD, Fidelman N, Gervais DA, Hohenwalter EJ, Kapoor BS, Kolbeck KJ, Kouri BE, Mansour MA, Nair AV, Rochon PJ, Shaw CM, Expert Panel on Interventional Radiology. . ACR Appropriateness Criteria® radiologic management of inferior vena cava filters. . 2012:10.

167. Kostelic JK RCJ, Lorenz JM, Burke CT, Darcy MD, Fidelman N, Hohenwalter EJ, Kinney TB, Kolbeck KJ, Kouri BE, Mansour MA, Nair AV, Owens CA, Rochon PJ, Vatakencherry G, Expert Panel on Interventional Radiology. . ACR Appropriateness Criteria® radiologic management of iliac artery occlusive disease. . 2012:8.

168. Kushi LH, Doyle C, McCullough M, et al. American Cancer Society Guidelines on nutrition and physical activity for cancer prevention: reducing the risk of cancer with healthy food choices and physical activity. *CA Cancer J Clin.* 2012;62(1):30-67.

169. Laine L, Jensen DM. Management of patients with ulcer bleeding. *Am J Gastroenterol.* 2012;107(3):345-360; quiz 361.

170. Lalani T, Couto CA, Rosen MP, et al. ACR appropriateness Criteria jaundice. *Journal of the American College of Radiology.* 2013;10(6):402-409.

171. Lally BE, Chang JY, Chetty IJ, et al. ACR Appropriateness Criteria® radiation therapy for small-cell lung cancer. *American journal of clinical oncology.* 2013;36(2):206-213.

172. Lamont J. Female sexual health consensus clinical guidelines. *J Obstet Gynaecol Can.* 2012;34(8):769-783.

173. Lane BF W-Y-CJ, Javitt MC, Glanc P, Brown DL, Dubinsky T, Harisinghani MG, Harris RD, Khati NJ, Mitchell DG, Pandharipande PV, Pannu HK, Podrasky AE, Shipp TD, Siegel CL, Simpson L, Wall DJ, Zelop CM, Expert Panel on Women's Imaging. . ACR Appropriateness Criteria® first trimester bleeding. 2012:7.

174. Lansberg MG, O'Donnell MJ, Khatri P, et al. Antithrombotic and thrombolytic therapy for ischemic stroke: Antithrombotic Therapy and Prevention of Thrombosis, 9th ed: American College of Chest Physicians Evidence-Based Clinical Practice Guidelines. *Chest.* 2012;141(2 Suppl):e601S-636S.

175. Lentine KL, Costa SP, Weir MR, et al. Cardiac disease evaluation and management among kidney and liver transplantation candidates: a scientific statement from the American Heart Association and the American College of Cardiology Foundation. *J Am Coll Cardiol.* 2012;60(5):434-480.

176. Leyendecker JR CM, Remer EM, Bishoff JT, Blaufox MD, Eberhardt SC, Friedman B, Hartman MS, Hosseinzadeh K, Lazarus E, Lockhart ME, Oto A, Porter C, Sudakoff GS, Verma S, Expert Panel on Urologic Imaging. . ACR Appropriateness Criteria® pretreatment staging of invasive bladder cancer. . 2012:12.

177. Lieberman DA, Rex DK, Winawer SJ, Giardiello FM, Johnson DA, Levin TR. Guidelines for colonoscopy surveillance after screening and polypectomy: a consensus update by the US Multi-Society Task Force on Colorectal Cancer. *Gastroenterology.* 2012;143(3):844-857.

178. Linkins LA, Dans AL, Moores LK, et al. Treatment and prevention of heparin-induced thrombocytopenia: Antithrombotic Therapy and Prevention of Thrombosis, 9th ed: American College of Chest Physicians Evidence-Based Clinical Practice Guidelines. *Chest.* 2012;141(2 Suppl):e495S-530S.

179. Lipsky BA, Berendt AR, Cornia PB, et al. 2012 Infectious Diseases Society of America clinical practice guideline for the diagnosis and treatment of diabetic foot infections. *Clin Infect Dis.* 2012;54(12):e132-173.

180. Lo SS LS, Chang EL, Galanopoulos N, Howell DD, Kim EY, Konski AA, Pandit-Taskar ND, Rose PS, Ryu S, Silverman LN, Sloan AE, Van Poznak C, Expert Panel on Radiation Oncology-Bone Metastases. . ACR Appropriateness Criteria® spinal bone metastases. . 2012:13.

181. Loutfy MR, Margolese S, Money DM, Gysler M, Hamilton S, Yudin MH. Canadian HIV pregnancy planning guidelines. *J Obstet Gynaecol Can.* 2012;34(6):575-590.

182. Luchs JS FJ, Weissman BN, Kransdorf MJ, Appel M, Arnold E, Bancroft LW, Bruno MA, Fries IB, Hayes CW, Jacobson JA, Morrison WB, Mosher TJ, Murphey MD, Palestro CJ, Roberts CC, Rubin DA, Tuite MJ, Ward RJ, Zoga AC, Expert Panel on Musculoskeletal Imaging. . ACR Appropriateness Criteria® chronic ankle pain. 2012:9.

183. Mack CL, Gonzalez-Peralta RP, Gupta N, et al. NASPGHAN practice guidelines: Diagnosis and management of hepatitis C infection in infants, children, and adolescents. *J Pediatr Gastroenterol Nutr.* 2012;54(6):838-855.

184. Mainiero MB, Lourenco A, Mahoney MC, et al. ACR Appropriateness Criteria breast cancer screening. *Journal of the American College of Radiology.* 2013;10(1):11-14.

185. Manchikanti L, Abdi S, Atluri S, et al. American Society of Interventional Pain Physicians (ASIPP) guidelines for responsible opioid prescribing in chronic non-cancer pain: Part 2--guidance. *Pain Physician.* 2012;15(3 Suppl):S67-116.

186. Marcus CL, Brooks LJ, Draper KA, et al. Diagnosis and management of childhood obstructive sleep apnea syndrome. *Pediatrics.* 2012;130(3):576-584.

187. Marino BS, Lipkin PH, Newburger JW, et al. Neurodevelopmental outcomes in children with congenital heart disease: evaluation and management: a scientific statement from the American Heart Association. *Circulation.* 2012;126(9):1143-1172.

188. McGillion M, Arthur HM, Cook A, et al. Management of patients with refractory angina: Canadian Cardiovascular Society/Canadian Pain Society joint guidelines. *Can J Cardiol.* 2012;28(2 Suppl):S20-41.

189. McMurray JJ, Adamopoulos S, Anker SD, et al. ESC Guidelines for the diagnosis and treatment of acute and chronic heart failure 2012: The Task Force for the Diagnosis and Treatment of Acute and Chronic Heart Failure 2012 of the European Society of Cardiology. Developed in collaboration with the Heart Failure Association (HFA) of the ESC. *Eur Heart J.* 2012;33(14):1787-1847.

190. Messenger AG, McKillop J, Farrant P, McDonagh AJ, Sladden M. British Association of Dermatologists' guidelines for the management of alopecia areata 2012. *Br J Dermatol.* 2012;166(5):916-926.

191. Meyer JS CB, Karmazyn B, Binkovitz LA, Dempsey-Robertson ME, Dillman JR, Dory CE, Garber M, Hayes LL, Keller MS, Kulkarni AV, Milla SS, Myseros JS, Paidas C, Widmann RF, Raske ME, Rigsby CK, Strouse PJ, Wootton-Gorges SL, Expert Panel on Pediatric Imaging. . ACR Appropriateness Criteria® suspected physical abuse -- child. 2012:9.

192. Milla SS, Coley BD, Karmazyn B, et al. ACR Appropriateness Criteria® Limping Child—Ages 0 to 5 Years. *Journal of the American College of Radiology.* 2012;9(8):545-553.

193. Miller KJ, Couchie C, Ehman W, Graves L, Grzybowski S, Medves J. Rural maternity care. *J Obstet Gynaecol Can.* 2012;34(10):984-1000.

194. Mitchell DG, Javitt MC, Glanc P, et al. ACR Appropriateness Criteria staging and follow-up of ovarian cancer. *Journal of the American College of Radiology.* 2013;10(11):822-827.

195. Monagle P, Chan AK, Goldenberg NA, et al. Antithrombotic therapy in neonates and children: Antithrombotic Therapy and Prevention of Thrombosis, 9th ed: American College of Chest Physicians Evidence-Based Clinical Practice Guidelines. *Chest.* 2012;141(2 Suppl):e737S-801S.

196. Montgomery A, Hale TW. ABM clinical protocol #15: analgesia and anesthesia for the breastfeeding mother, revised 2012. *Breastfeed Med.* 2012;7(6):547-553.

197. Moyer VA. Screening for ovarian cancer: U.S. Preventive Services Task Force reaffirmation recommendation statement. *Ann Intern Med.* 2012;157(12):900-904.

198. Moyer VA. Screening for hearing loss in older adults: U.S. Preventive Services Task Force recommendation statement. *Ann Intern Med.* 2012;157(9):655-661.

199. Moyer VA. Screening for chronic kidney disease: U.S. Preventive Services Task Force recommendation statement. *Ann Intern Med.* 2012;157(8):567-570.

200. Moyer VA. Screening for coronary heart disease with electrocardiography: U.S. Preventive Services Task Force recommendation statement. *Ann Intern Med.* 2012;157(7):512-518.

201. Moyer VA. Behavioral counseling interventions to promote a healthful diet and physical activity for cardiovascular disease prevention in adults: U.S. Preventive Services Task Force recommendation statement. *Ann Intern Med.* 2012;157(5):367-371.

202. Moyer VA. Screening for and management of obesity in adults: U.S. Preventive Services Task Force recommendation statement. *Ann Intern Med.* 2012;157(5):373-378.

203. Moyer VA. Prevention of falls in community-dwelling older adults: U.S. Preventive Services Task Force recommendation statement. *Ann Intern Med.* 2012;157(3):197-204.

204. Moyer VA. Screening for prostate cancer: U.S. Preventive Services Task Force recommendation statement. *Ann Intern Med.* 2012;157(2):120-134.

205. Moyer VA. Behavioral counseling to prevent skin cancer: U.S. Preventive Services Task Force recommendation statement. *Ann Intern Med.* 2012;157(1):59-65.

206. Moyer VA. Screening for cervical cancer: U.S. Preventive Services Task Force recommendation statement. *Ann Intern Med.* 2012;156(12):880-891, w312.

207. Moyer VA. Menopausal hormone therapy for the primary prevention of chronic conditions: U.S. Preventive Services Task Force recommendation statement. *Ann Intern Med.* 2013;158(1):47-54.

208. Muayqil T, Gronseth G, Camicioli R. Evidence-based guideline: diagnostic accuracy of CSF 14-3-3 protein in sporadic Creutzfeldt-Jakob disease: report of the guideline development subcommittee of the American Academy of Neurology. *Neurology.* 2012;79(14):1499-1506.

209. Nakhleh RE, Myers JL, Allen TC, et al. Consensus statement on effective communication of urgent diagnoses and significant, unexpected diagnoses in surgical pathology and cytopathology from the College of American Pathologists and Association of Directors of Anatomic and Surgical Pathology. *Arch Pathol Lab Med.* 2012;136(2):148-154.

210. Nikolaidis P CD, Remer EM, Bishoff JT, Coursey CA, Dighe M, Eberhardt SC, Goldfarb S, Harvin HJ, Lazarus E, Leyendecker JR, Lockhart ME, Majd M, Oto A, Porter C, Ramchandani P, Sheth S, Vikram R, Expert Panel on Urologic Imaging. ACR Appropriateness Criteria® acute pyelonephritis. 2012:6.

211. Nuwer MR, Emerson RG, Galloway G, et al. Evidence-based guideline update: intraoperative spinal monitoring with somatosensory and transcranial electrical motor evoked potentials: report of the Therapeutics and Technology Assessment Subcommittee of the American Academy of Neurology and the American Clinical Neurophysiology Society. *Neurology.* 2012;78(8):585-589.

212. Oliva IB, Davarpanah AH, Rybicki FJ, et al. ACR appropriateness criteria® imaging of mesenteric ischemia. *Abdominal imaging.* 2013;38(4):714-719.

213. Oto A YJ, Casalino DD, Remer EM, Blaufox MD, Bishoff JT, Coursey CA, Dighe M, Eberhardt SC, Harvin HJ, Lazarus E, Leyendecker JR, Lockhart ME, Nikolaidis P, Porter C, Ramchandani P, Sheth S, Vikram R, Expert Panel on Urologic Imaging. . ACR Appropriateness Criteria® staging of testicular malignancy. . 2012:7.

214. Pandharipande PV HH, Javitt MC, Glanc P, Bennett GL, Brown DL, Dubinsky T, Harisinghani MG, Harris RD, Horowitz NS, Mitchell DG, Pannu HK, Podrasky AE, Shipp TD, Siegel CL, Simpson L, Wong-You-Cheong JJ, Zelop CM, Expert Panel on Women’s Imaging. . ACR Appropriateness Criteria® ovarian cancer screening. . 2012:6.

215. Panel on Treatment of HIV-Infected Pregnant Women and Prevention of Perinatal Transmission. Recommendations for use of antiretroviral drugs in pregnant HIV-1-infected women for maternal health and interventions to reduce perinatal HIV transmission in the United States. . 2014:Various.

216. Panel. AAoOPOS. Amblyopia. . 2012:39.

217. Patwa HS, Chaudhry V, Katzberg H, Rae-Grant AD, So YT. Evidence-based guideline: intravenous immunoglobulin in the treatment of neuromuscular disorders: report of the Therapeutics and Technology Assessment Subcommittee of the American Academy of Neurology. *Neurology.* 2012;78(13):1009-1015.

218. Perinatal PoToH-IPWaPo, Transmission. Recommendations for Use of Antiretroviral Drugs in Pregnant HIV-1-

Infected Women for Maternal Health and Interventions to Reduce Perinatal HIV

Transmission in the United States. . 2012. <http://aidsinfo.nih.gov/contentfiles/lvguidelines/PerinatalGL.pdf>.

219. Perk J, De Backer G, Gohlke H, et al. European Guidelines on cardiovascular disease prevention in clinical practice (version 2012). The Fifth Joint Task Force of the European Society of Cardiology and Other Societies on Cardiovascular Disease Prevention in Clinical Practice (constituted by representatives of nine societies and by invited experts). *Eur Heart J.* 2012;33(13):1635-1701.

220. Pottie K, Jaramillo A, Lewin G, et al. Recommendations on screening for type 2 diabetes in adults. *Cmaj.* 2012;184(15):1687-1696.

221. Pringsheim T, Davenport W, Mackie G, et al. Canadian Headache Society guideline for migraine prophylaxis. *Can J Neurol Sci.* 2012;39(2 Suppl 2):S1-59.

222. Qaseem A, Denberg TD, Hopkins RH, Jr., et al. Screening for colorectal cancer: a guidance statement from the American College of Physicians. *Ann Intern Med.* 2012;156(5):378-386.

223. Qaseem A, Fihn SD, Dallas P, Williams S, Owens DK, Shekelle P. Management of stable ischemic heart disease: summary of a clinical practice guideline from the American College of Physicians/American College of Cardiology Foundation/American Heart Association/American Association for Thoracic Surgery/Preventive Cardiovascular Nurses Association/Society of Thoracic Surgeons. *Ann Intern Med.* 2012;157(10):735-743.

224. Qaseem A, Fihn SD, Williams S, Dallas P, Owens DK, Shekelle P. Diagnosis of stable ischemic heart disease: summary of a clinical practice guideline from the American College of Physicians/American College of Cardiology Foundation/American Heart Association/American Association for Thoracic Surgery/Preventive Cardiovascular Nurses Association/Society of Thoracic Surgeons. *Ann Intern Med.* 2012;157(10):729-734.

225. Qaseem A, Humphrey LL, Sweet DE, Starkey M, Shekelle P. Oral pharmacologic treatment of type 2 diabetes mellitus: a clinical practice guideline from the American College of Physicians. *Ann Intern Med.* 2012;156(3):218-231.

226. Quality AU-AfHRa. Practice parameter for psychodynamic psychotherapy with children. American Academy of Child and Adolescent Psychiatry. . 2012.

227. Quality AU-AfHRa. Guidelines for the use of antiretroviral agents in pediatric HIV infection. . 2012.

228. Quality AU-AfHRa. ACR Appropriateness Criteria® nonpalpable mammographic findings (excluding calcifications). . 2012.

229. Ray CE, Lorenz JM, Burke CT, et al. ACR Appropriateness Criteria Radiologic Management of Benign and Malignant Biliary Obstruction. *Journal of the American College of Radiology.* 2013;10(8):567-574.

230. Reilly JT, McMullin MF, Beer PA, et al. Guideline for the diagnosis and management of myelofibrosis. *Br J Haematol.* 2012;158(4):453-471.

231. Remer EM CD, Bishoff JT, Coursey CA, Dighe M, Eberhardt SC, Goldfarb S, Harvin HJ, Lazarus E, Leyendecker JR, Lockhart ME, Majd M, Nikolaidis P, Oto A, Porter C, Ramchandani P, Sheth S, Vikram R, Expert Panel on Urologic Imaging. . ACR Appropriateness Criteria® incidentally discovered adrenal mass. 2012:10.

232. Restrepo RD, Hirst KR, Wittnebel L, Wettstein R. AARC clinical practice guideline: transcutaneous monitoring of carbon dioxide and oxygen: 2012. *Respir Care.* 2012;57(11):1955-1962.

233. Restrepo RD, Walsh BK. Humidification during invasive and noninvasive mechanical ventilation: 2012. *Respir Care.* 2012;57(5):782-788.

234. Ridge JA LJ, Beitler JJ, Yom SS, Garg MK, McDonald MW, Quon H, Saba N, Salama JK, Smith RV, Worden F, Yeung AR, Expert Panel on Radiation Oncology-Head & Neck Cancer. . ACR Appropriateness Criteria® treatment of stage I T1 glottic cancer. . 2012:8.

235. Roberts CC WB, Appel M, Bancroft LW, Bennett DL, Bruno MA, Fries IB, Germano I, Hayes CW, Holly L, Jacobson JA, Kransdorf MJ, Luchs JS, Morrison WB, Mosher TJ, Murphey MD, Palestro CJ, Rubin DA, Stoller DW, Tuite MJ, Ward RJ, Wise JN, Zoga AC, Lutz ST, Expert Panel on Musculoskeletal Imaging. ACR Appropriateness Criteria®: Metastatic bone disease. 2012:12.

236. Rochon PJ VC, Ray CE Jr, Lorenz JM, Burke CT, Darcy MD, Hohenwalter EJ, Kinney TB, Kolbeck KJ, Kostelic JK, Kouri BE, Mansour MA, Nair AV, Owens CA, Vatakencherry G, Expert Panel on Interventional Radiology. . ACR Appropriateness Criteria® radiologic management of lower-extremity venous insufficiency. . 2012:9.

237. Rock CL, Doyle C, Demark-Wahnefried W, et al. Nutrition and physical activity guidelines for cancer survivors. *CA Cancer J Clin.* 2012;62(4):243-274.

238. Rosenzweig KE CJ, Chetty IJ, Decker RH, Ginsburg ME, Kestin LL, Kong FM, Lally BE, Langer CJ, Movsas B, Videtic GMM, Willers H, Expert Panel on Radiation Oncology-Lung. . ACR Appropriateness Criteria® nonsurgical treatment for non-small-cell lung cancer: poor performance status or palliative intent. . 2012:13.

239. Rubin DA WB, Appel M, Arnold E, Bencardino JT, Fries IB, Hayes CW, Hochman MG, Jacobson JA, Luchs JS, Math KR, Murphey MD, Newman JS, Scharf SC, Small KM, Expert Panel on Musculoskeletal Imaging. . ACR Appropriateness Criteria® chronic wrist pain. . 2012:13.

240. Rupp SM, Apfelbaum JL, Blitt C, et al. Practice guidelines for central venous access: a report by the American Society of Anesthesiologists Task Force on Central Venous Access. *Anesthesiology.* 2012;116(3):539-573.

241. Saad W, Al-Osaimi AM, Caldwell S, Ray CE Jr, Lorenz JM, Burke CT, Darcy MD, Fidelman N, Greene FL, Hohenwalter EJ, Kinney TB, Kolbeck KJ, Kostselic JK, Kouri BE, Nair AV, Rochon PJ, Rockey DC, Vatakencherry G, Expert Panel on Interventional Radiology. ACR Appropriateness Criteria® radiologic management of gastric varices. 2012:8.

242. Saslow D, Solomon D, Lawson HW, et al. American Cancer Society, American Society for Colposcopy and Cervical Pathology, and American Society for Clinical Pathology screening guidelines for the prevention and early detection of cervical cancer. *CA Cancer J Clin.* 2012;62(3):147-172.

243. Schenker MP RF, Dill KE, Desjardins B, Flamm SD, Francois CJ, Gerhard-Herman MD, Kalva SP, Mansour MA, Mohler ER III, Oliva IB, Weiss C, Expert Panel on Vascular Imaging. . ACR Appropriateness Criteria® recurrent symptoms following lower extremity angioplasty. . 2012:7.

244. Schweitzer ME, Daffner RH, Weissman BN, et al. ACR Appropriateness Criteria® on Suspected Osteomyelitis in Patients With Diabetes Mellitus. *Journal of the American College of Radiology.* 2012;5(8):881-886.

245. Scully M, Hunt BJ, Benjamin S, et al. Guidelines on the diagnosis and management of thrombotic thrombocytopenic purpura and other thrombotic microangiopathies. *Br J Haematol.* 2012;158(3):323-335.

246. Shaffer WO BJ, Fernand R, Matz P. . Antibiotic prophylaxis in spine surgery. . 2012:72.

247. Sharlip ID, Belker AM, Honig S, et al. Vasectomy: AUA guideline. *The Journal of urology.* 2012;188(6 Suppl):2482-2491.

248. Shergill AK, Ben-Menachem T, Chandrasekhara V, et al. Guidelines for endoscopy in pregnant and lactating women. *Gastrointest Endosc.* 2012;76(1):18-24.

249. Sheth S CD, Remer EM, Bishoff JT, Coursey CA, Dighe M, Eberhardt SC, Goldfarb S, Harvin HJ, Lazarus E, Leyendecker JR, Lockhart ME, Nikolaidis P, Oto A, Porter C, Vikram R, Expert Panel on Urologic Imaging. . ACR Appropriateness Criteria® renal trauma. . 2012:7.

250. Shrim A, Koren G, Yudin MH, Farine D. Management of varicella infection (chickenpox) in pregnancy. *J Obstet Gynaecol Can.* 2012;34(3):287-292.

251. Shulman ST, Bisno AL, Clegg HW, et al. Clinical practice guideline for the diagnosis and management of group A streptococcal pharyngitis: 2012 update by the Infectious Diseases Society of America. *Clin Infect Dis.* 2012;55(10):e86-102.

252. Silberstein SD, Holland S, Freitag F, Dodick DW, Argoff C, Ashman E. Evidence-based guideline update: pharmacologic treatment for episodic migraine prevention in adults: report of the Quality Standards Subcommittee of the American Academy of Neurology and the American Headache Society. *Neurology.* 2012;78(17):1337-1345.

253. Singh JA, Furst DE, Bharat A, et al. 2012 update of the 2008 American College of Rheumatology recommendations for the use of disease-modifying antirheumatic drugs and biologic agents in the treatment of rheumatoid arthritis. *Arthritis Care Res (Hoboken).* 2012;64(5):625-639.

254. Smallridge RC, Ain KB, Asa SL, et al. American Thyroid Association guidelines for management of patients with anaplastic thyroid cancer. *Thyroid.* 2012;22(11):1104-1139.

255. Smith BD, Morgan RL, Beckett GA, et al. Recommendations for the identification of chronic hepatitis C virus infection among persons born during 1945-1965. *MMWR Recomm Rep.* 2012;61(Rr-4):1-32.

256. Society. NAS. Diagnosis and treatment of lumbar disc herniation with radiculopathy. . 2012:100.

257. Srivastava A, Brewer AK, Mauser‐Bunschoten EP, et al. Guidelines for the management of hemophilia. *Haemophilia.* 2013;19(1):e1-e47.

258. Stachler RJ, Chandrasekhar SS, Archer SM, et al. Clinical practice guideline: sudden hearing loss. *Otolaryngol Head Neck Surg.* 2012;146(3 Suppl):S1-35.

259. Steele SR, Varma MG, Melton GB, Ross HM, Rafferty JF, Buie WD. Practice parameters for anal squamous neoplasms. *Dis Colon Rectum.* 2012;55(7):735-749.

260. Steg PG, James SK, Atar D, et al. ESC Guidelines for the management of acute myocardial infarction in patients presenting with ST-segment elevation. *Eur Heart J.* 2012;33(20):2569-2619.

261. Steiner I, Schmutzhard E, Sellner J, Chaudhuri A, Kennedy PG. EFNS-ENS guidelines for the use of PCR technology for the diagnosis of infections of the nervous system. *Eur J Neurol.* 2012;19(10):1278-1291.

262. Sudakoff GS RM, Rybicki FJ, Blake MA, Cash BD, Desjardins B, Greene FL, Hindman NM, Oliva IB, Weiss C, Yaghmai V, Expert Panels on Vascular Imaging and Gastrointestinal Imaging. ACR Appropriateness Criteria® blunt abdominal trauma. 2012:9.

263. Thompson MA, Mugavero MJ, Amico KR, et al. Guidelines for improving entry into and retention in care and antiretroviral adherence for persons with HIV: evidence-based recommendations from an International Association of Physicians in AIDS Care panel. *Ann Intern Med.* 2012;156(11):817-833, w-284, w-285, w-286, w-287, w-288, w-289, w-290, w-291, w-292, w-293, w-294.

264. Tinegate H, Birchall J, Gray A, et al. Guideline on the investigation and management of acute transfusion reactions. Prepared by the BCSH Blood Transfusion Task Force. *Br J Haematol.* 2012;159(2):143-153.

265. Tsao MN, Rades D, Wirth A, et al. Radiotherapeutic and surgical management for newly diagnosed brain metastasis (es): An American Society for Radiation Oncology evidence-based guideline. *Practical radiation oncology.* 2012;2(3):210-225.

266. Vahanian A, Alfieri O, Andreotti F, et al. Guidelines on the management of valvular heart disease (version 2012). *Eur Heart J.* 2012;33(19):2451-2496.

267. Van Eyk N, van Schalkwyk J. Antibiotic prophylaxis in gynaecologic procedures. *J Obstet Gynaecol Can.* 2012;34(4):382-391.

268. Vandvik PO, Lincoff AM, Gore JM, et al. Primary and secondary prevention of cardiovascular disease: Antithrombotic Therapy and Prevention of Thrombosis, 9th ed: American College of Chest Physicians Evidence-Based Clinical Practice Guidelines. *Chest.* 2012;141(2 Suppl):e637S-668S.

269. Vassil AD VG, Gore EM, Bradley JD, Buatti JM, Germano I, Ghafoori AP, Henderson MA, Lutz ST, Murad GJA, Patchell RA, Patel SH, Robbins JR, Robins HI, Wippold FJ II, Yunes MJ, Expert Panel on Radiation Oncology-Brain Metastasis. . ACR Appropriateness Criteria® single brain metastasis. . 2012:9.

270. Venning VA, Taghipour K, Mohd Mustapa MF, Highet AS, Kirtschig G. British Association of Dermatologists' guidelines for the management of bullous pemphigoid 2012. *Br J Dermatol.* 2012;167(6):1200-1214.

271. Vos PE, Alekseenko Y, Battistin L, et al. Mild traumatic brain injury. *Eur J Neurol.* 2012;19(2):191-198.

272. Watson H, Davidson S, Keeling D. Guidelines on the diagnosis and management of heparin-induced thrombocytopenia: second edition. *Br J Haematol.* 2012;159(5):528-540.

273. Watts NB, Adler RA, Bilezikian JP, et al. Osteoporosis in men: an Endocrine Society clinical practice guideline. *J Clin Endocrinol Metab.* 2012;97(6):1802-1822.

274. Weiss C AE, Rybicki FJ, Kim HS, Desjardins B, Fan CM, Flamm SD, Francois CJ, Gerhard-Herman MD, Kalva SP, Mansour MA, Mohler ER III, Oliva IB, Schenker MP, Expert Panel on Vascular Imaging. . ACR Appropriateness Criteria® sudden onset of cold, painful leg. . 2012:6.

275. Weissman SM, Burt R, Church J, et al. Identification of individuals at risk for Lynch syndrome using targeted evaluations and genetic testing: National Society of Genetic Counselors and the Collaborative Group of the Americas on Inherited Colorectal Cancer joint practice guideline. *J Genet Couns.* 2012;21(4):484-493.

276. Wespes E HK, Eardley I, Guiliano F, Hatzichristou D, Moncada I, Salonia A, Vardi Y. . Guidelines on penile curvature. . 2012:24.

277. Whitlock RP, Sun JC, Fremes SE, Rubens FD, Teoh KH. Antithrombotic and thrombolytic therapy for valvular disease: Antithrombotic Therapy and Prevention of Thrombosis, 9th ed: American College of Chest Physicians Evidence-Based Clinical Practice Guidelines. *Chest.* 2012;141(2 Suppl):e576S-600S.

278. Winters JC, Dmochowski RR, Goldman HB, et al. Urodynamic studies in adults: AUA/SUFU guideline. *The Journal of urology.* 2012;188(6 Suppl):2464-2472.

279. Wippold FJ CR, Aiken AH, Amin-Hanjani S, Berger KL, Broderick DF, Davis PC, Douglas AC, Hoh BL, Mechtler LL, Smirniotopoulos JG, Expert Panel on Neurologic Imaging. ACR Appropriateness Criteria® focal neurologic deficit. 2012:11.

280. Wippold FJ II CR, Berger KL, Broderick DF, Davis PC, Douglas AC, Frey KA, Mechtler LL, Nussenbaum B, Smirniotopoulos JG, Expert Panel on Neurologic Imaging. . ACR Appropriateness Criteria® neck mass/adenopathy. . 2012:9.

281. Wippold FJ II CR, Berger KL, Broderick DF, Davis PC, Douglas AC, Germano IM, Hadley JA, McDermott MW, Mechtler LL, Smirniotopoulos JG, Waxman AD, Expert Panel on Neurologic Imaging. . ACR Appropriateness Criteria® orbits, vision and visual loss. [online publication]. . 2012:12.

282. Wippold FJ II CR, Aiken AH, Angevine PD, Angtuaco EJ, Brown DC, Fries IB, Holly L, McConnell CT Jr, Mechtler LL, Roth CJ, Seidenwurm DJ, Waxman AD, Winfree CJ, Expert Panel on Neurologic Imaging. . ACR Appropriateness Criteria® plexopathy. . 2012:14.

283. Wippold FJ II CR, Aiken AH, Angtuaco EJ, Berger KL, Brown DC, Davis PC, Holloway K, McConnell CT Jr, Mechtler LL, Nussenbaum B, Rosenow JM, Roth CJ, Seidenwurm DJ, Slavin K, Waxman AD, Expert Panel on Neurologic Imaging. . ACR Appropriateness Criteria® cranial neuropathy. 2012:18.

284. Wong SL, Balch CM, Hurley P, et al. Sentinel lymph node biopsy for melanoma: American Society of Clinical Oncology and Society of Surgical Oncology joint clinical practice guideline. *J Clin Oncol.* 2012;30(23):2912-2918.

285. Woodard PK, White RD, Abbara S, et al. ACR Appropriateness Criteria chronic chest pain—low to intermediate probability of coronary artery disease. *Journal of the American College of Radiology.* 2013;10(5):329-334.

286. You JJ, Singer DE, Howard PA, et al. Antithrombotic therapy for atrial fibrillation: Antithrombotic Therapy and Prevention of Thrombosis, 9th ed: American College of Chest Physicians Evidence-Based Clinical Practice Guidelines. *Chest.* 2012;141(2 Suppl):e531S-575S.

287. Zelop CM JM, Glanc P, Dubinsky T, Harisinghani MG, Harris RD, Khati NJ, Mitchell DG, Pandharipande PV, Pannu HK, Podrasky AE, Shipp TD, Siegel CL, Simpson L, Wall DJ, Wong-You-Cheong JJ, Expert Panel on Women's Imaging. . ACR Appropriateness Criteria® growth disturbances -- risk of intrauterine growth restriction. . 2012:7.

288. Zerey M, Hawver LM, Awad Z, et al. SAGES evidence-based guidelines for the laparoscopic resection of curable colon and rectal cancer. *Surgical endoscopy.* 2013;27(1):1-10.

289. Zoga AC WB, Kransdorf MJ, Adler R, Appel M, Bancroft LW, Bruno MA, Fries IB, Morrison WB, Mosher TJ, Palestro CJ, Roberts CC, Tuite MJ, Ward RJ, Expert Panel on Musculoskeletal Imaging. . ACR Appropriateness Criteria® soft-tissue masses. . 2012:8.

290. АНЕМІЇ К. KDIGO clinical practice guideline for anemia in chronic kidney disease. *Kidney International.* 2012;2:279.
