# Supplementary material for: Financial Relationships between Organizations That Produce Clinical Practice Guidelines and the Biomedical Industry: A Cross-Sectional Study
Source: PLoS Med. 2016 May 31;13(5):e1002029. doi: 10.1371/journal.pmed.1002029 (PMC4887051; doi:10.1371/journal.pmed.1002029)
Supplement: S4 Text — (DOCX) [file pmed.1002029.s005.docx]

**S4 Text: Organizations producing clinical practice guidelines**

| **Organization** | **Website** |
| --- | --- |
| Academy of breast feeding medicine | <http://www.bfmed.org> |
| American college of physicians | <https://www.acponline.org> |
| American academy of child and adolescent psychiatry | <http://www.aacap.org> |
| American academy of family physicians | <http://www.aafp.org> |
| American academy of neurology | <https://www.aan.com> |
| American academy of ophthalmology | <http://www.aao.org> |
| American academy of orthopaedic surgeons (AAOS) | <http://www.aaos.org> |
| American academy of otolaryngology - head and neck surgery foundation | <http://www.entnet.org> |
| American academy of pediatrics | <https://www.aap.org> |
| American academy of sleep medicine | <http://www.aasmnet.org> |
| American association of neuromuscular and electrodiagnostic medicine | <https://www.aanem.org> |
| American association of respiratory care | <http://www.aarc.org> |
| American association for the study of liver disease | <http://www.aasld.org> |
| American association of thoracic surgery | <http://www.aats.org> |
| American association of clinical endrocrinologists | <https://www.aace.com> |
| American cancer society | <http://www.cancer.org> |
| American clinical neurophysiology society | <http://www.acns.org> |
| American college of cardiology | <http://www.acc.org> |
| American college of chest physicians | <https://www.chestnet.org> |
| American college of emergency physicians | <http://www.acep.org> |
| American college of gastroenterology | <http://gi.org> |
| American college of obstetricians and gynecologists | <http://www.acog.org> |
| American college of radiology | <http://www.acr.org> |
| American college of rheumatology | <http://www.rheumatology.org> |
| American diabetes association | <http://www.diabetes.org> |
| American gastroenterological association institute | <http://www.gastro.org> |
| American geriatrics society | <http://www.americangeriatrics.org> |
| American heart association | <http://www.heart.org/> |
| American society for clinical pathology | <http://www.ascp.org> |
| American society for colposcopy and cervical pathology | <http://www.asccp.org> |
| American society of clinical oncology | <http://www.asco.org> |
| American Society of Colon and Rectal Surgeons | <https://www.fascrs.org> |
| American society of echocardiography | <http://asecho.org> |
| American society of gastrointestinal endoscopy | <http://www.asge.org> |
| American society for radiation oncology | <https://www.astro.org> |
| American society of anesthesiologists | <https://www.asahq.org> |
| American society of interventional pain physicians | <https://asipp.org> |
| American society of parenteral and enteral nutrition | <https://www.nutritioncare.org> |
| American thyroid association | <http://www.thyroid.org> |
| American urological association | <https://www.auanet.org> |
| Association of directors of anatomic and surgical pathology | <http://www.adasp.org> |
| British association of dermatologists | <http://www.bad.org.uk> |
| British association of sexual health and HIV | <http://www.bashh.org> |
| British Committee for standards in hematology | <http://www.bcshguidelines.com> |
| British society for bone marrow transplantation | <http://bsbmt.org> |
| British thoracic society | <https://www.brit-thoracic.org.uk> |
| Canadian agency for drugs and technologies in health | <https://www.cadth.ca> |
| Canadian cardiovascular society | <http://www.ccs.ca/en/> |
| Canadian headache society | <http://headachenetwork.ca> |
| Canadian pain society | <http://www.canadianpainsociety.ca> |
| Canadian task force on preventative health care | <http://canadiantaskforce.ca> |
| Center for disease control | <http://www.cdc.gov> |
| Chartered institute of environmental health | <http://www.cieh.org> |
| Child neurology society | <http://www.childneurologysociety.org> |
| Collaborative group of the Americas on inherited colorectal cancer joint practice guideline | <http://www.cgaicc.com> |
| College of American pathologists | <http://www.cap.org> |
| Endocrine society | <https://www.endocrine.org> |
| European association of cardio-thoracic surgery | <http://www.eacts.org> |
| European federation of neurological sciences | <https://www.eaneurology.org> |
| European association of urology | <https://uroweb.org> |
| European heart rhythm association | <http://www.escardio.org/The-ESC/Communities/European-Heart-Rhythm-Association-(EHRA)/EHRA> |
| European cardiac arrhythmia society | <http://ecas-heartrhythm.org> |
| European society of cardiology | <https://www.escardio.org> |
| Faculty of sexual and reproductive health care | <http://www.fsrh.org> |
| Health protection agency | <https://www.gov.uk/government/organisations/public-health-england> |
| Heart failure society of America | <http://www.hfsa.org> |
| Heart rhythm society | <http://www.hrsonline.org> |
| Infectious disease society of America | <http://www.idsociety.org> |
| International association for chronic fatigue syndrome/myalgic encephalomyelitis | <http://iacfsme.org> |
| International association of physicians in AIDS care | <http://www.iapac.org> |
| International league against epilepsy | <http://www.ilae.org> |
| Kidney disease: improving global outcomes | <http://kdigo.org/home/> |
| Kidney disease outcomes quality initiative (national kidney foundation) | <https://www.kidney.org> |
| National Collaborating Centre for Women's and Children's Health | <http://www.ncc-wch.org.uk> |
| National collaborating centre for cancer | <http://www.wales.nhs.uk/sites3/home.cfm?orgid=432> |
| Neurocritical Care Society | <http://www.neurocriticalcare.org> |
| National Institute for health and care excellence | <https://www.nice.org.uk> |
| North American menopause society | <http://www.menopause.org> |
| North American society for pediatric gastroenterology, hepatology and nutrition | <http://www.naspghan.org> |
| North American spine society | <https://www.spine.org> |
| Office of AIDS research advisory council | <http://www.oar.nih.gov/oarac/> |
| Pediatric and congenital electrophysiology society | <http://pediatricepsociety.org> |
| Royal Australian college of general practitioners | <http://www.racgp.org.au> |
| Royal college of obstetricians and gynaecologists | <https://www.rcog.org.uk> |
| Scottish intercollegiate guideline network | <http://www.sign.ac.uk> |
| Society for cardiovascular angiography and interventions | <http://www.scai.org> |
| Society of American gastrointestinal and endoscopic surgeons | <http://www.sages.org> |
| Society of critical care medicine | <http://www.sccm.org> |
| Society of obstetricians and gynaecologists of Canada | <http://sogc.org> |
| Society of surgical oncology | <http://www.surgonc.org> |
| Society of thoracic surgeons | <http://www.sts.org> |
| Society of urodymics, female pelvic medicine and urogenital reconstruction | <http://sufuorg.com> |
| US Preventative Services Task Force | <http://www.uspreventiveservicestaskforce.org> |
| World federation of hemophilia | <https://www.wfh.org> |
