# Supplementary material for: Financial Relationships between Organizations That Produce Clinical Practice Guidelines and the Biomedical Industry: A Cross-Sectional Study
Source: PLoS Med. 2016 May 31;13(5):e1002029. doi: 10.1371/journal.pmed.1002029 (PMC4887051; doi:10.1371/journal.pmed.1002029)
Supplement: S1 STROBE — (DOC) [file pmed.1002029.s006.doc]

STROBE Statement—checklist of items that should be included in reports of observational studies

|  | Item No | Recommendation |
| --- | --- | --- |
| **Title and abstract** | 1 | (*a*) Indicate the study’s design with a commonly used term in the title or the abstract  Financial Relationships Between Organizations that Produce Clinical Practice Guidelines and the Biomedical Industry: a Cross Sectional Study |
|  |  | (*b*) Provide in the abstract an informative and balanced summary of what was done and what was found  Abstract, paragraph 3 |
| Introduction | | |
| Background/rationale | 2 | Explain the scientific background and rationale for the investigation being reported  Introduction, paragraphs 1-2 |
| Objectives | 3 | State specific objectives, including any prespecified hypotheses  Introduction, paragraph 2 |
| Methods | | |
| Study design | 4 | Present key elements of study design early in the paper  Introduction, paragraph 2 |
| Setting | 5 | Describe the setting, locations, and relevant dates, including periods of recruitment, exposure, follow-up, and data collection  Methods, paragraphs 2-7 |
| Participants | 6 | (*a*) *Cohort study*—Give the eligibility criteria, and the sources and methods of selection of participants. Describe methods of follow-up  *Case-control study*—Give the eligibility criteria, and the sources and methods of case ascertainment and control selection. Give the rationale for the choice of cases and controls  *Cross-sectional study*—Give the eligibility criteria, and the sources and methods of selection of participants  Methods, paragraphs 2-7 |
|  |  | (*b*) *Cohort study*—For matched studies, give matching criteria and number of exposed and unexposed  Not Applicable  *Case-control study*—For matched studies, give matching criteria and the number of controls per case  Not Applicable |
| Variables | 7 | Clearly define all outcomes, exposures, predictors, potential confounders, and effect modifiers. Give diagnostic criteria, if applicable  Methods, paragraphs 4-6 |
| Data sources/ measurement | 8* | For each variable of interest, give sources of data and details of methods of assessment (measurement). Describe comparability of assessment methods if there is more than one group  Methods, paragraphs 4-6 |
| Bias | 9 | Describe any efforts to address potential sources of bias  Discussion, paragraph 6 |
| Study size | 10 | Explain how the study size was arrived at  Methods, paragraph 2 |
| Quantitative variables | 11 | Explain how quantitative variables were handled in the analyses. If applicable, describe which groupings were chosen and why  Methods, paragraph 8. |
| Statistical methods | 12 | (*a*) Describe all statistical methods, including those used to control for confounding  Methods, paragraph 8. |
|  |  | (*b*) Describe any methods used to examine subgroups and interactions  Not Applicable |
|  |  | (*c*) Explain how missing data were addressed  Methods, paragraph 8. |
|  |  | (*d*) *Cohort study*—If applicable, explain how loss to follow-up was addressed  *Case-control study*—If applicable, explain how matching of cases and controls was addressed  *Cross-sectional study*—If applicable, describe analytical methods taking account of sampling strategy  Not Applicable |
|  |  | (*e*) Describe any sensitivity analyses  Not Applicable |

Continued on next page

| Results | | |
| --- | --- | --- |
| Participants | 13* | (a) Report numbers of individuals at each stage of study—eg numbers potentially eligible, examined for eligibility, confirmed eligible, included in the study, completing follow-up, and analysed  Figure 1 |
|  |  | (b) Give reasons for non-participation at each stage  Figure 1 |
|  |  | (c) Consider use of a flow diagram  Figure 1 |
| Descriptive data | 14* | (a) Give characteristics of study participants (eg demographic, clinical, social) and information on exposures and potential confounders  Table 1 |
|  |  | (b) Indicate number of participants with missing data for each variable of interest  Table 1 |
|  |  | (c) *Cohort study*—Summarise follow-up time (eg, average and total amount)  Not Applicable |
| Outcome data | 15* | *Cohort study*—Report numbers of outcome events or summary measures over time  Not Applicable |
|  |  | *Case-control study—*Report numbers in each exposure category, or summary measures of exposure  Not Applicable |
|  |  | *Cross-sectional study—*Report numbers of outcome events or summary measures  Tables 2-4 |
| Main results | 16 | (*a*) Give unadjusted estimates and, if applicable, confounder-adjusted estimates and their precision (eg, 95% confidence interval). Make clear which confounders were adjusted for and why they were included  Tables 2-4 |
|  |  | (*b*) Report category boundaries when continuous variables were categorized  Not Applicable |
|  |  | (*c*) If relevant, consider translating estimates of relative risk into absolute risk for a meaningful time period  Not Applicable |
| Other analyses | 17 | Report other analyses done—eg analyses of subgroups and interactions, and sensitivity analyses  Not Applicable |
| Discussion | | |
| Key results | 18 | Summarise key results with reference to study objectives  Discussion, paragraph 1 |
| Limitations | 19 | Discuss limitations of the study, taking into account sources of potential bias or imprecision. Discuss both direction and magnitude of any potential bias  Discussion, paragraph 6 |
| Interpretation | 20 | Give a cautious overall interpretation of results considering objectives, limitations, multiplicity of analyses, results from similar studies, and other relevant evidence  Discussion, paragraphs 1-7 |
| Generalisability | 21 | Discuss the generalisability (external validity) of the study results  Discussion, paragraph 6 |
| Other information | | |
| Funding | 22 | Give the source of funding and the role of the funders for the present study and, if applicable, for the original study on which the present article is based  Funding Statement |

*Give information separately for cases and controls in case-control studies and, if applicable, for exposed and unexposed groups in cohort and cross-sectional studies.

**Note:** An Explanation and Elaboration article discusses each checklist item and gives methodological background and published examples of transparent reporting. The STROBE checklist is best used in conjunction with this article (freely available on the Web sites of PLoS Medicine at http://www.plosmedicine.org/, Annals of Internal Medicine at http://www.annals.org/, and Epidemiology at http://www.epidem.com/). Information on the STROBE Initiative is available at www.strobe-statement.org.
